# Ecotype-associated structural variants across multiple locally adapted Poeciliid fishes

**DOI:** 10.1186/s12864-026-13205-9

**Published:** 2026-08-01

**Authors:** Kara Ryan, Rishi De-Kayne, Lenin Arias-Rodriguez, Michael Tobler, Joanna L. Kelley

**Affiliations:** 1 Department of Ecology and Evolutionary Biology, University of California Santa Cruz, Santa Cruz, CA 95060 USA; 2 División Académica de Ciencias Biológicas, Universidad Juárez Autónoma de Tabasco (UJAT), Villahermosa, México; 3 Department of Biology, University of Missouri—St. Louis, St. Louis, MO 63121 USA; 4 Whitney R. Harris World Ecology Center, University of Missouri—St. Louis, St. Louis, MO 63121 USA; 5 WildCare Institute, Saint Louis Zoo, St. Louis, MO 63110 USA

**Keywords:** Adaptation, Extreme environments, Convergent evolution, Poecillidae, Structural variation, Pangenome

## Abstract

**Background:**

Extreme environments provide powerful natural laboratories for studying evolutionary convergence across taxa. While convergent phenotypes frequently evolve in response to similar selective pressures, consistent molecular signatures at the nucleotide level are often elusive, potentially due to overlooked genetic variants such as structural variants. Here, we leverage recent advances in long-read sequencing and pangenome approaches to investigate the role of structural variants in repeated adaptation to toxic springs rich in hydrogen sulfide (H_2_S) across multiple independently evolved populations within the *Poecilia mexicana* species complex.

**Results:**

We identified more than 99,000 structural variants over 50 base pairs in length, including insertions, deletions, duplications and inversions, using complementary graph- and read-based methods. We then integrated these with population genomic data and RNA-sequencing data to detect variants in highly differentiated regions between ecotypes and assess their functional impacts. Structural variants in both coding and regulatory regions were enriched in genes involved in sulfide metabolism and ion transport, including *ethe1*, *slc13a1*, and *sqor*. We found ecotype-specific structural variants in promoter and intronic regions of key detoxification genes that exhibited elevated expression in H_2_S-adapted populations.

**Conclusions:**

Our findings suggest that structural variation may contribute to adaptive phenotypes and should be considered in studies of genomic convergence.

**Supplementary Information:**

The online version contains supplementary material available at https://doi.org/10.1186/s12864-026-13205-9.

## Introduction 

Extreme environments provide striking examples of convergent evolution across taxa, suggesting a limited set of evolutionary solutions. These environments serve as natural experiments, offering powerful frameworks to investigate convergent evolution across biological scales [1]. Strong selective pressures frequently drive the independent evolution of similar phenotypes—from molecular function to behavior—across lineages. For example, in subterranean habitats, the combination of darkness and nutrient scarcity results in a suite of convergent traits, such as loss of pigment and eyes, across distantly related taxa [2–5]. In some cases, phenotypic convergence is underpinned by repeated de novo mutations in the same genes or even identical amino acid positions (e.g. *prestin* in echolocating bats and dolphins [6]). However, many convergent traits do not exhibit such repeated, locus-specific sequence changes across taxa [7]. Similar phenotypes often arise without parallel mutations in the same genes or sites, and the underlying genetic changes may occur at different loci or remain undetected at the sequence level. In these cases, convergence often occurs at higher levels of biological organization, such as within gene regulatory networks, biological pathways, or functions [8], even in extreme environments [9, 10].

Most genomic studies of convergent evolution have focused on single nucleotide polymorphisms (SNPs), potentially overlooking other forms of genetic variation. Structural variants—including insertions, deletions, duplications, and inversions—represent additional forms of genomic variation that are abundant genome-wide but have been historically difficult to confidently identify and genotype, despite their ability to affect gene regulation and protein function. For example, structural variants have been identified as a major driver of gene expression differences among human populations [11] and can shape functional differences in proteins, often through disordered conformations [12, 13]. The absence of consistent sequence-level signals across taxa could reflect either the existence of multiple genomic solutions to similar selective pressures or a bias in investigating SNPs over structural variants. Therefore, incorporating structural variants into studies of genomic convergence is critical for a comprehensive understanding of the genomic architecture underpinning adaptive traits.

Structural variants have been recognized as important in evolutionary processes for over a century [14, 15] and can directly influence adaptation and speciation [16–18]. They underpin a broad range of phenotypes, including variation in coloration and color patterning [19, 20], morphology [21], and behavior [22, 23]. In fishes, for example, copy number variation in the *fads2* gene is associated with dietary differences in stickleback [24], and chromosomal inversions help maintain migratory and resident ecotypes in Atlantic cod [25]. Across distantly related taxa, ranging from primates to butterflies, independent gene duplication events of opsin genes have repeatedly led to enhanced color vision [26, 27]. However, structural variants have historically been underrepresented in evolutionary genomic studies due to technical limitations in detecting them with short-read sequencing and alignment-based methods, especially in repetitive or structurally complex regions. The increasing accessibility of long-read sequencing has renewed interest in structural variants across a wider range of taxa [17, 28, 29]. Genomic patterns of diversity inferred by structural variants can differ from those estimated from SNPs [30], and structural variants’ roles in evolutionary processes, such as adaptation and speciation, should be studied directly [16, 31]. Despite increasing recognition of their evolutionary significance, few studies have comprehensively examined the role of structural variants in repeated adaptation across independently evolved populations.

In the livebearing fish family Poeciliidae, multiple lineages have independently adapted to toxic springs rich in hydrogen sulfide evolving convergent phenotypes in life history, morphology, physiology, and gene expression [32–37]. Despite convergence across levels of organization, evidence for convergence at the gene and nucleotide level is limited [32, 38, 39]. Furthermore, where signals of selection on similar regions have been detected, they appear to be primarily driven by selection on shared, standing variation and not independent de novo mutations [40]. Importantly, these previous studies have focused mainly on SNP variation, leaving the contribution of structural variants largely unexplored, although one study identified a few shared structural variants among sulfide-adapted populations of *Poecilia mexicana* [41]. These were identified despite using short reads (100 base pair, bp), a highly fragmented reference genome, and a small sample size (N = 1 per population), which limited the ability to detect variants and downstream analyses. Recent advances, including high-quality reference assemblies [42] and improved long- and short-read sequencing technologies [43, 44], enable a more comprehensive analysis of structural variants, their potential role in repeated adaptation to sulfide springs in *P. mexicana*, and the extent to which adaptive structural variation represents a common feature of repeated adaptation in closely related lineages.

In this study, we investigate the role of structural variants in repeated adaptation to sulfide springs in *Poecilia mexicana*. Using Pacific Biosciences (PacBio) HiFi long read data from three individuals, two from sulfidic environments and one from nonsulfidic, we assembled pseudo-haplotype-resolved, contig-level genomes and constructed a *P. mexicana* pangenome graph. We then applied both graph- and long-read based structural variant detection approaches to comprehensively identify variants 50 bp or longer. We then generated population-level, whole-genome resequencing data from five sulfidic and five closely related, nonsulfidic populations to assess variation in the frequency of structural variants among populations and identify highly differentiated SNPs. Finally, we integrated previously published RNA-sequencing (RNA-seq) data from [33] and [45] to assess overlap between differentially expressed genes and structural variants in noncoding regions. By integrating SNPs, structural variants, and gene expression data from multiple sulfidic and nonsulfidic populations of *P. mexicana*, this work offers new insights into the genomic architecture of convergence in extreme environments and addresses broader questions about the predictability of evolution at the molecular level.

## Methods

### Focal populations

We analyzed ten populations of the *Poecilia mexicana* species complex distributed across four river drainages in the Río Grijalva basin of Chiapas and Tabasco, Mexico: Pichulcalo (Pich), Ixtapangajoya (Ixta), Puyacatengo (Puya), and Tacotalpa (Taco). These included five sulfide-spring (S) populations and five nonsulfidic (NS) populations. Sulfidic populations consisted of two *P. mexicana* populations (Taco S, Puya S), two *Poecilia sulphuraria* populations (Pich1 S, Pich2 S), and one *Poecilia thermalis* population (Ixta S). The closely related NS populations included five *P. mexicana* populations (Taco NS, Puya NS, Ixta NS, Pich1 NS, Pich2 NS) (Table S1).

Fish were caught with a seine (2 × 4 m). Immediately after capture, individual fish were sacrificed using an overdose of buffered MS222 (Tricaine methanesulfonate) and measured and weighed for a separate study. We sampled a fin clip from each fish using sterilized scissors and forceps. Fin clips were preserved in 2 ml of RNAlater (Ambion, Inc.). DNA was extracted from muscle tissue using the Gentra Puregene Tissue Kit (Qiagen) according to the manufacturer’s protocol for purifying genomic DNA from 5–10 mg of tissue, with the following modifications: tissue homogenization was performed using a micropestle; centrifugation after addition of the protein precipitation solution was extended to 3.5 min; and centrifugation after addition of isopropanol was conducted for 2 min. Extracted DNA was quantified with a Qubit fluorometer and its integrity verified by electrophoresis on a 1% agarose gel.

### Identification of structural variants using long reads

We identified structural variants using previously generated PacBio HiFi reads [42] from three focal populations: Taco S, Pich2 S, and Taco NS (Bioproject PRJNA984711). Briefly, DNA was extracted using the Circulomics Nanobind High Molecular Weight DNA extraction kit. Quantity was assessed using the Qubit™ High Sensitivity DNA kit in triplicate, Quantus™ Fluorometer, and Nanodrop and quality was assessed using an Agilent™ Femto Pulse system and TapeStation. SMRTbell library preparation and sequencing was performed at UC Davis Genome Center on a single SMRT cell on a PacBio Revio sequencing platform.

Reads were mapped to the *P. mexicana* genome assembly (GCA_051168715.1; [42] using pbmm2 (https://github.com/PacificBiosciences/pbmm2), a wrapper for minimap2 [46]. Variants were called per sample using Sawfish v2.0.1 [47] *discovery* followed by joint genotyping with *joint-call*. The resulting Variant Call Format (VCF) files were filtered to remove low-quality calls (QUAL < 10), excessive depth (defined as > 12 times the GC-corrected haploid-depth estimate, representing the expected mean genome-wide depth per haploid copy), redundant breakpoints already represented in the VCF, and conflicting multi-breakpoint genotypes flagged by sawfish. Additionally, we used Sniffles2 v2.6.1 [48] to generate an intermediate sniffles (SNF) file for each individual and performed multi-sample variant calling to generate a VCF file. Variants were filtered using BCFtools v1.21 [49] to retain high quality variants (QUAL ≥ 30) and to exclude imprecise variants (bcftools view -e 'INFO/IMPRECISE'), homozygous reference calls (bcftools view -i 'GT[*] = "alt"'), and break-end variants (bcftools view -i 'INFO/SVTYPE = "BND"').

### Pangenome generation, variant calling

To generate a pangenome graph representative of assembled haplotypes, we used Hifiasm 0.25.0-r726 [50] with default parameters to generate contig-level, pseudo-haplotype-resolved assemblies for each individual with pre-existing HiFi data (N = 3). FASTA headers were renamed to conform to the PanSN-spec: Pangenome Sequence Naming standard (https://github.com/pangenome/PanSN-spec). A reference-guided pangenome was constructed using the Minigraph-Cactus pipeline [51]. First, a structural variant graph (rGFA) was generated using Minigraph v0.20-r599 [52], with the chromosome level *P. mexicana* NS assembly (GCA_051168715.1; [42]) as the backbone. Haplotype assemblies were mapped back to graph using Minigraph, and a base-pair-level multiple genome alignment was generated using Cactus.

The final pangenome graph was then prepared for downstream analysis using the VG toolkit [53] *view* and *index* functions. Variants were extracted using VG *deconstruct* and the resulting VCF was filtered using VCFbub [54] to remove nested sites unless nested under variants greater than 10 kilobases (kb). We then annotated variant types using Truvari v5.3.0 [55] *anno svinfo.*

### Structural variant merging across calling methods

Each structural variant callset (Sniffles2, Sawfish and graph-derived) was normalized using BCFtools *norm* to left-align indels, correct reference alleles, and decompose multiallelic records. Structural variants within each multi-sample VCF were then collapsed using Truvari v5.3.0 *collapse*, which clusters structural variants that are similar in position and size and retains a single representative variant per cluster, keeping the highest-quality call (with parameters -r 500 -p 0.95 -P 0.95 -S 100000 -s 50 -k maxqual --passonly). To generate a nonredundant merged set of variants from long-read methods, we used Jasmine v1.1.5 [56]. Duplications were temporarily converted to insertions (--preprocess_only --dups_to_ins) to reduce merging errors. Variants were merged and redundant variants across methods were removed (max_dist = 500 –nonlinear-distance). After merging, duplications were restored to their original variant type and variants with unknown variant type were filtered out. To merge across read and graph-based approaches, we first normalized and removed redundant calls from the graph-based method using BCFtools and Truvari as described above. Then, variants were merged between graph and read based methods using SURVIVOR [57] v1.0.7.

### Short-read library preparation and sequencing

Whole-genome sequencing Illumina libraries were prepared from 80 individuals (8 per population) previously sampled for capture sequencing [40]. Genomic DNA, stored at –80 °C for approximately six years, was thawed and quantified using Qubit the high-sensitivity DNA kit. Samples, chosen to optimize DNA quality and quantity, were shipped overnight on ice to the Genomics and Cell Characterization Core Facility (GC3F) at the University of Oregon. At GC3F, 10 ng of DNA per sample was dried in a SpeedVac in a 384-well plate and 6 µl of a diluted Bead-Linked Transposase (Illumina) (1:60 in buffer: 10 mM Tris–HCL, 5 mM MgCl_2_, 8% PEG) was added. The samples were vortexed to resuspend the DNA and incubated at 53 °C for 30 min. After the plate was spun down and placed on a magnet to gather beads, 5 µl of supernatant was removed. Then, 1 µl of barcoded Illumina Nextera adapter primers (IDT), 5 µl 2 × Equinox Master-mix (Watchmaker Genomics), and 3 µl Nuclease Free Water (NEB) were added to each well. PCR was conducted as follows: 68 °C for 3 min, 98 °C for 3 min, 10 cycles of 98 °C for 45 s, 62 °C for 30 s, 68 °C for 2 min, followed by final extension at 68 °C for 1 min. After amplification, 20 µl of nuclease free water was added to each well and mixed. A 2 µl aliquot per sample was pooled and purified twice with 0.8 × volume cleaning with Total Pure NGS beads (Omega Biosciences). The pooled library was run on Illumina MiSeq Nano PE150. Based on read count normalization, a rebalanced pool was sequenced on two lanes of an Illumina NovaSeq S4 (PE150).

### Identification of highly differentiated regions

Read quality was assessed using FastQC v0.12.1 [58] and trimmed using fastp v0.32.2 [59]. Trimmed reads were mapped to the *P. mexicana* reference genome (GCA_051168715.1) using bwa-mem2 [60]. The resulting SAM files were converted to BAM and sorted and indexed using Sambamba [61] v0.8.2. Single nucleotide polymorphisms (SNPs) were called using BCFtools *mpileup* (--skip-indels) and *call*. BCFtools *view* was used to filter bialleleic SNPs (-m2 -M2 -v snps). The resulting SNPs were further filtered using VCFtools [62] for quality (--minQ 30), missingness (--max-missing 0.9), depth (--min-meanDP 3 --max-meanDP 250) and minor allele count (--mac 8). Retained SNPs were then thinned to retain 1 SNP per 1 kb to reduce the effects of linkage disequilibrium. We then used pixy [63] to calculate the average Weir and Cockerham F_ST_ [64] for 10-kb windows across the genome. We converted windowed F_ST_ values to Z-score and defined outlier regions based on a 0.99 F_ST_ empirical threshold (F_ST_ > 0.431) and Z-score (> 2.996).

### Population level genotyping of structural variants

To estimate allele frequencies of structural variants, we used GraphTyper2 v2.7.7 [65] with the genotype_sv module to genotype the previously identified variants using the short-read BAM files generated above. To do so, we modified the merged VCF file to anchor the reference sequence to the first position of the insertion sequence for graph generation. For genotyping, the default minimum coverage filter (10X) was disabled to account for lower genome-wide sequencing depth across individuals. Aggregate genotype calls per individual were determined by the highest genotype quality across different genotyping models, including both coverage and breakpoint-based models. The subsequent genotyped VCFs were merged using BCFtools. Allele counts and frequencies were then calculated with BCFtools separately for sulfidic and nonsulfidic groups. We retained variants that were genotyped in at least 20 individuals per ecotype. Finally, allele frequency differences between sulfidic and nonsulfidic populations were calculated in R by subtracting the allele frequency in the non-sulfidic population from the frequency in the sulfidic population.

### Analysis of structural variants and gene expression

We imported structural variants into R using VariantAnnotation v1.54.1 and GenomicRanges v.1.60.0. The GFF annotation file was imported, and features were extracted using rtracklayer v.1.68.0. Promoters were defined as the 1-kb window upstream of the transcription start site (strand-aware) and introns were defined as gene regions excluding exons using *setdiff* and *reduce*. Then, overlaps between highly differentiated regions (F_ST_ outlier windows), genomic features, and structural variants were identified using *findoverlaps().*

Integrative Genomics Viewer (IGV) was used to visualize variants of interest in long and short read alignments. Gene Ontology (GO) enrichment was performed with ClusterProfiler v4.16.0, with GO terms considered significantly enriched if Benjamini–Hochberg adjusted *p*-value was less than 0.05.

We reanalyzed RNA-seq count data from gill tissue focusing on both field-collected samples [33] as well as samples from a nonsulfidic common-garden H_2_S exposure experiment, that included both laboratory-control and H_2_S exposure groups from two population pairs of sulfidic and nonsulfidic populations (Tacotalpa and Puyacatengo, n = 4–6 per group) [45]. Using EdgeR4.4.2 [89], we filtered low-count genes (filterByExpr), calculated normalization factors (calcNormFactors) and estimated tagwise dispersion (estimateTagwiseDisp). We modeled differential expression using glmFit and tested contrasts between ecotypes using glmLRT. Differentially expressed genes (Benjamini–Hochberg adjusted eR v*p*-value < 0.05) were then intersected with small variants (50–1000 bp) present in promoters and intron regions.

To assess whether overlap between structural variants and differentially expressed genes exceeded random expectations, we performed a permutation analysis in R. Gene sets of equal size to the observed differentially expressed gene set were randomly sampled from the set of all annotated genes (10,000 iterations), and the number of genes overlapping structural variants was recalculated for each iteration to generate an empirical null distribution. The empirical *p*-value was calculated as the proportion of permutations with equal or greater overlap than observed.

### Generation of gene trees

Given evidence for differential expression between ecotypes, we investigated sequence divergence and evolutionary relationships of two duplicate copies of *ethe1* across populations. In order to explore variation among populations, we generated gene trees for both copies of *ethe1*. First, we downloaded the RefSeq proteins of both copies of *ethe1* from NCBI for *Gambusia affinis*, *Xiphophorus helleri, Poecilia reticulata*, *Poecilia mexicana*, *Fundulus heteroclitus*, and for single copy *ethe1* for *Danio rerio*. We then used mafft v.7.526 [90] to align nucleotide gene sequences. From there, we used Iqtree2 v.2.2.6 [91] to generate a maximum likelihood tree with 1,000 ultrafast bootstrap replicates. We then generated consensus sequences for both *ethe1* gene regions from the VCF file generated above for each *P. mexicana* individual. The headers in the resulting consensus sequences were renamed to include the sample name and then concatenated. These resulting fasta files were then aligned using mafft and a maximum likelihood tree with 1,000 ultrafast bootstrap replicates was generated for the *P. mexicana* individuals. Trees were visualized in R using ggtree v3.16.3, treeio v1.32.0, and ape v.5.8.1.

## Results

### Structural variant discovery with long reads

To characterize structural variation, we analyzed long-read sequencing data from three individuals representing two sulfidic populations (*P. sulphuraria* and *P. mexicana* S Taco) and one nonsulfidic population (*P. mexicana* NS Taco), with an estimated coverage of 22-32X and a median read length of 10,263—11,005 base pairs (bp) (Table S1; [42]). These long-read datasets were used for complementary reference-based structural variant discovery (Sawfish and Sniffles2) and for pangenome-based variant detection. Then, these structural variants were merged and filtered to result in a set of variants for downstream population-level genotyping. Of note, structural variant discovery was based on three individuals, and therefore likely represents a subset of the total structural variation present across populations. As a result, rare or population-specific variants may be underrepresented in the discovery set.

After filtering and collapsing redundant structural variants within each method, Sawfish detected 114,017 variants (53,417 deletions, 60,213 insertions, 185 inversions, and 202 duplications), while Sniffles2 identified 86,212 variants (40,524 deletions, 45,371 insertions, 260 inversions, 57 duplications) across three individuals with long read sequencing data [42]. Merging these variants and reducing redundancy in calls resulted in 93,759 unique variants (43,586 deletions, 49,599 insertions, 340 inversions and 234 duplications). Pangenome generation and structural variant calling was performed using haplotype-resolved assemblies (113–405 haplotypes per individual; Table S2) identified 69,409 variants (33,198 deletions, 35,911 insertions, and 300 variants of unknown type). Merging graph-based variant calls with the long-read based call set resulted in 99,016 variants (45,581 deletions, 52,893 insertions, 308 inversions, and 234 duplications) (Supplementary Fig. 1 A). Variant length ranged from 50 bp to over 10 megabases (Mb), with distinct distributions of sizes observed across SV types (Supplementary Fig. 1B). Median length was 244 bp for deletions, 383 bp for insertions, 2,699 bp for inversions, and 18,788 bp for duplications.

### Genotyping of structural variants and variation in allele frequencies between ecotypes

To quantify allele frequency variation between habitats, we generated short read sequencing data from eight individuals per population from 10 populations of the *Poecilia mexicana* species complex (five sulfidic and five nonsulfidic). Whole-genome short-read sequencing of 80 individuals across ten populations produced an average of 26.8 million reads per individual across two sequencing lanes (range: 16.9–56.6 million reads) and a mean estimated coverage of 11.71X (9.44–21.19X; Table S3). Using these data, we genotyped structural variants identified from long-read sequencing to assess differences in allele frequencies between ecotypes. After filtering, we retained 39,496 structural variants (50 bp to > 5 Mb), comprising 35,068 deletions, 2,423 insertions, 2,995 duplications, and 10 inversions. Genotyping success varied by SV type, with deletions and duplications genotyped in the majority of samples (median ~ 80 individuals), whereas inversions (median ~ 70 individuals) and insertions (median ~ 58 individuals) showed lower and more variable genotyping rates. Although insertions were well represented at the discovery stage, they were substantially underrepresented after population-level genotyping; consequently, downstream analyses are primarily driven by deletions and duplications, which were more reliably genotyped across individuals. Population-genotyped variants showed a shift toward smaller sizes for deletions and insertions (median 166 bp and 144 bp, respectively) compared to the full discovery set (244 bp and 383 bp), whereas duplications and inversions had similar median sizes between datasets. We then identified the most differentiated variants between ecotypes (top 1% difference in allele frequency; Supplementary Fig. 2, Table S4), which consisted of 3 deletions, 2 duplications, and 3 insertions. The mean size of these highly differentiated variants was 781 bp (median = 264 bp), and 31 overlapped annotated gene regions (Table S4). The most differentiated variant was a 419-bp deletion in an intron of ethe1a (AF_S = 1.0, AF_NS = 0.15). The relatively small number of insertions among genotyped variants is consistent with reduced sensitivity for detecting and genotyping insertions relative to other SV types using short-read data [43, 66].

### Single nucleotide polymorphism detection and identification of highly differentiated regions

To identify highly differentiated regions between ecotypes, we identified genome-wide single nucleotide polymorphisms (SNPs). After filtering, we retained 578,183 SNPs. Genome-wide average F_ST_ between sulfide and nonsulfidic populations was 0.136. We identified highly differentiated genomic windows (10 kb, F_ST_ > 0.431, Z-score > 2.996) as outliers that are putatively under selection (Supplementary Fig. 3). Outlier windows overlapped with 823 genes (668 unique genes, Table S5).

### Analysis of large and mid-sized structural variants

To identify candidate structural variants potentially underlying adaptation to sulfide springs, we first examined large variants (> 50 kb) due to their ability to influence large genomic regions and potentially many genes at once. To avoid potentially spurious SV calls, we excluded variants exceeding 5 Mb. Large variants consisted of 54 deletions, 70 insertions, 32 inversions, and 8 duplications (Supplementary Fig. 4). Two noteworthy large variants overlapped genomic regions identified as highly differentiated based on SNP-based F_ST_: a deletion on scaffold 12 and an inversion on scaffold 6.

**Fig. 1:**
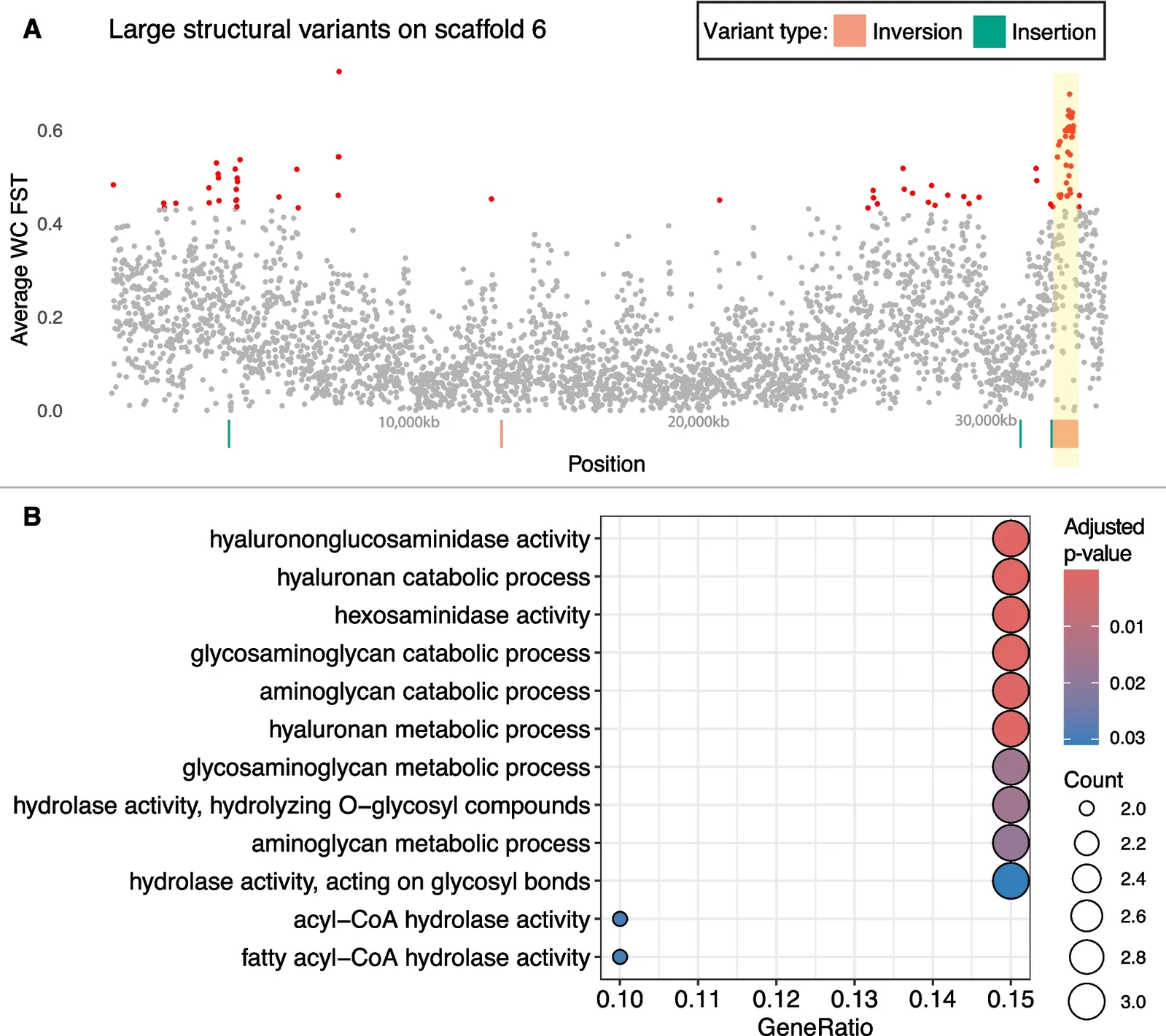
Large inversion highly differentiated between ecotypes. **A** Average Weir and Cockerham F_ST_ (WC F_ST_) between all sulfidic and nonsulfidic individuals in 10-kb windows. Outliers with Z score > 2.996 and a F_ST_ > 0.431 (0.99 empirical cutoff) are colored red. Structural variants greater than 50 kb are plotted below the axis. Inversions are plotted in orange and insertions are plotted in green. **B** Gene Ontology (GO) enrichment analysis of genes in the inversion highlighted in yellow in (A). All significantly enriched (Benjamini Hochberg corrected *p*-value < 0.05) GO terms are plotted

The 3.6-Mb deletion on scaffold 12 affected 140 genes, including N-acetylglucosamine-6-sulfatase (gns) and 2-oxoglutarate dehydrogenase (ogdh), but did not have significant enrichment of any biological processes or functions. This variant was at low frequency in both sulfidic (0.0375) and nonsulfidic (0.0625) populations, suggesting it is unlikely to contribute to adaptation despite occurring within a highly differentiated region (Table S6). The inversion on scaffold 6 encompassed 33 genes, 27 of which had functional annotation information (Fig. 1A) including two copies of slc13a1, a Na+-sulfate cotransporter gene involved in sulfate homeostasis, and hyal4 (hyaluronidase-4), which modifies the structure of chondroitin sulphate. Gene Ontology (GO) analysis of the genes in the inversion on scaffold 6 identified significant enrichment for biological processes and molecular functions related to hyaluronan catabolism (GO:0030214, GO:0030212) and glycosaminoglycan metabolic processes (GO:0006027, GO:0030203, GO:0006026, GO:0006022) (Fig. 1B). These variants were significantly enriched in genes involved in a broad range of transmembrane transport activities, with enrichment driven by eight genes overlapping structural variants (*foxj1*, *runx1*, *stat5a*, *slc4a8*, *slc13a1*, *slc26a2*, *slc18a1*, and DKK1, many of which are shared across GO terms (Figure 1B). However, we were unable to genotype this variant across individuals to assess its allele frequency distribution between ecotypes.

We identified 1,117 unique medium sized structural variants (10–50 kb), consisting of 492 deletions, 560 insertions, 60 duplications, and 5 inversions. Scaffold 18 was a hotspot for variants of this size (118 variants), whereas all other scaffolds contained fewer than 76 variants each (Supplementary Fig. 5). Of these medium sized variants, 25 variants occurred within SNP-based highly differentiated regions and overlapped 17 annotated genes. Some of these genes are involved in oxidative phosphorylation (OxPhos) complex assembly, including NADH dehydrogenase [ubiquinone] 1 alpha subcomplex assembly factor 5 (*ndufaf5*) (involved in Complex I assembly) and protein PET117 cytochrome c oxidase chaperone (*pet117*). However, these variants did not consistently show strong allele frequency differentiation between ecotypes and GO enrichment analysis did not reveal significant functional enrichment, suggesting that patterns at these genes could be driven by SNP-based divergence rather than structural variation.

Two variants were at high frequency in sulfidic populations (AF > 0.94) and low to mid frequency in nonsulfidic populations (AF < 0.34): a 13.8-kb deletion of LOC106932973 (CUB and zona pellucida-like domain-containing protein 1) and a 13.1-kb deletion that did not overlap any gene (Table S4). Additionally, three variants were at low frequency in sulfidic populations (< 0.15) and mid to high frequency in nonsulfidic populations (> 0.56), including an 11.7-kb deletion that affects *slc22a13*, a 19.6-kb deletion that affects dihydropyrimidinase-related protein 2, rac GTPase-activating protein 1, and protein phosphatase 1 regulatory subunit 3C-B, and a 13.9-kb deletion that did not overlap any genes.

**Fig. 2:**
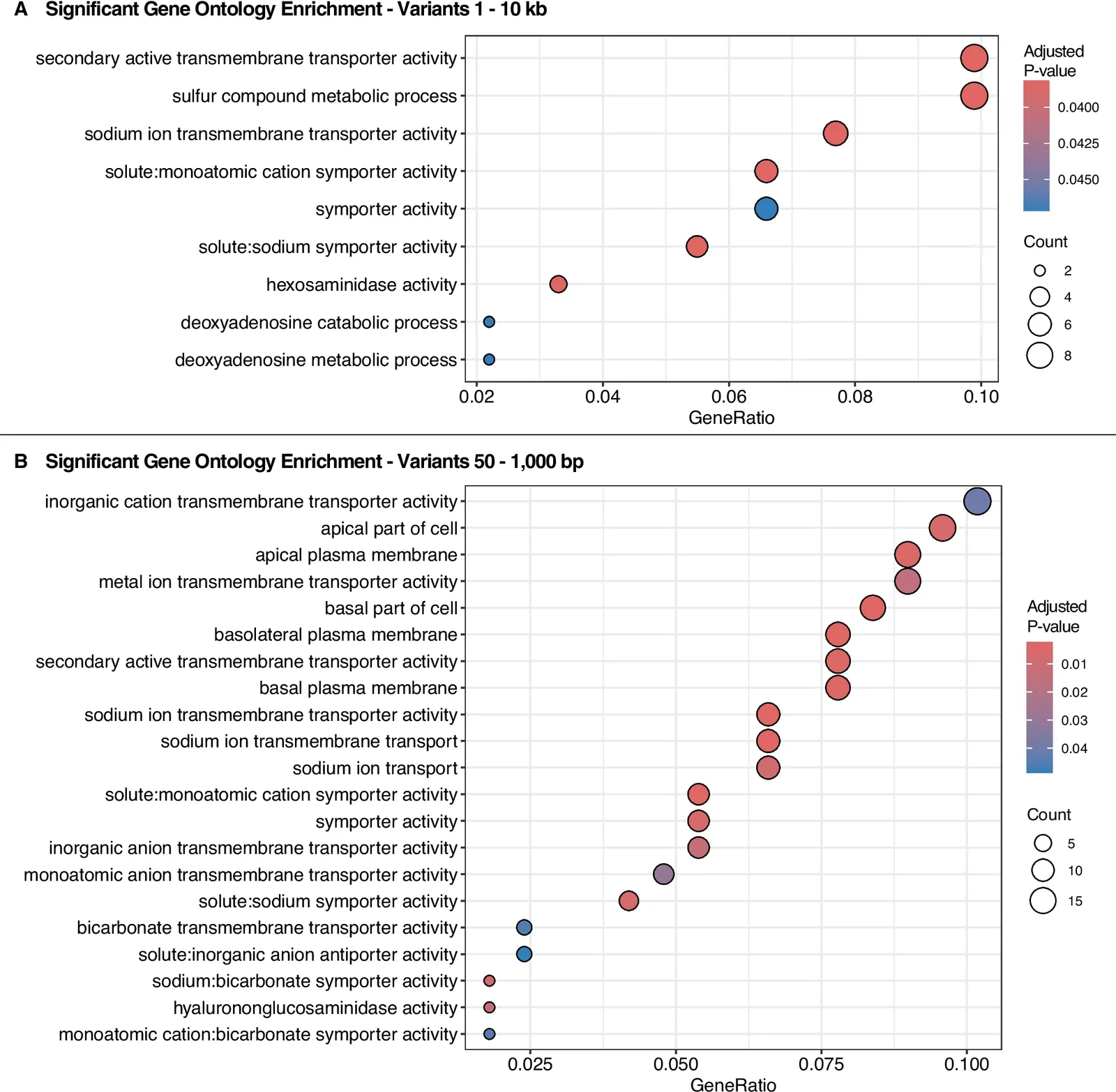
Gene ontology (GO) terms that are significantly enriched in variants (**A**) 1–10 kb or (**B**) 50–1,000 bp and in regions highly differentiated between ecotypes. All significant terms (Benjamini Hochberg adjusted *p*-value < 0.05) were plotted

Next, we analyzed structural variants 1–10 kb in size. These variants consisted of 8,344 deletions, 9,912 insertions, and 102 duplications. Although most variants occurred in intergenic regions, 124 genes overlapped with variants of this size, of which 94 genes had functional annotations. A subset of these variants occurred within genomic regions identified as highly differentiated based on SNP-based FST, indicating overlap between structural variation and sequence-based divergence. These genes included persulfide dioxygenase (ethe1a and ethe1b) and sulfide quinone oxidoreductase (sqor). Genes overlapping these variants were significantly enriched in a biological process (sulfur compound metabolic process; GO:0006790) and molecular functions secondary active transmembrane transporter activity (GO:0015291), sodium ion transmembrane transporter activity (GO:0015081), solute: monoatomic cation symporter activity (GO:0015294), hexosaminidase activity (GO:0015929) and solute: sodium symporter activity (GO:0015370) (Fig. 2A).

Some of these structural variants also showed allele frequency differences between ecotypes. For example, a 2.3-kb deletion in sqor differed substantially in frequency between sulfidic (AF = 0.025) and nonsulfidic populations (AF = 0.590). In total, 21 variants had significantly higher allele frequencies in sulfidic populations than nonsulfidic populations, affecting 8 genes, whereas 40 variants had lower allele frequencies in sulfidic populations, affecting 17 genes (Table S4).

### Analysis of variants shorter than 1 kb

Most structural variants were 50 bp to 1 kb in size, with 33,995 deletions, 33,437 insertions and 99 inversions called in this size range. Of these ~ 68,000 small structural variants, 29,284 overlapped with annotated gene regions. Among these, 181 genes overlapped both small structural variants and genomic regions identified as highly differentiated between ecotypes based on SNP-based F_ST_. These genes included several that localize to the mitochondria and are involved in sulfide metabolism, including 2-oxoglutarate dehydrogenase (*ogdh*), persulfide dioxygenase (*ethe1*), sulfide:quinone oxidoreductase (*sqor*), pyruvate carboxylase (*pc*), and alpha-aminoadipic semialdehyde synthase (*aass*). Across all genes overlapping small structural variants, we detected significant enrichment for biological processes related to transmembrane transport and ion exchange, including sodium ion transport (GO:0015081, GO:0035725, GO:0006814), metal ion transport (GO:0046873), and bicarbonate or inorganic anion exchange (GO:0015106, GO:0005452) (Fig. 2B). These transporters spanned diverse molecular functions such as symporters, antiporters, and secondary active transporters (e.g., GO:0015291, GO:0015294, GO:0015103), and were enriched in localization to specialized regions of the plasma membrane (GO:0016323, GO:0009925, GO:0016324).

To assess whether this enrichment was specific to genes within highly differentiated genomic regions, we compared GO enrichment for genes overlapping small structural variants within SNP-F_ST_ outlier windows to all genes in SNP-F_ST_ outlier regions. Neither gene set showed significant enrichment after multiple-testing correction, indicating that the observed functional enrichment is not restricted to, nor enhanced within, highly differentiated regions, but instead reflects broader patterns among genes affected by small structural variants.

Given the potential functional and regulatory impacts of these variants, we further investigated variants in this size class coding, putative promoter, and intronic regions in more detail. Among variants that overlapped with genes, 2,558 variants overlapped with exons, with 1,924 variants affecting protein-coding sequences. These coding-region variants included 996 deletions, 927 insertions, and one inversion. The mean variant length was 224 bp and the median was 129 bp. Exon-overlapping variants were more frequently in-frame (44.4% of variants) than expected based on the genome-wide distribution for this size class (35.9%) (Supplementary Fig. 6). Of the 2,558 exon-overlapping variants, 40 had allele-frequency estimates after genotyping. None showed high allele-frequency differentiation between ecotypes (AF difference ≥ 0.5), and none were among the top 1% most differentiated variants, suggesting that these coding-region variants are unlikely to represent major differentiated SVs between ecotypes.

### Associations of noncoding structural variants and differentially expressed genes

Regulatory changes have been shown to underlie adaptive divergence between sulfidic and nonsulfidic populations [32, 33, 45, 67, 68]. To investigate how structural variants in non-coding regions, specifically introns and promoters, relate to differential expression, we reanalyzed RNA-seq data generated by Kelley (2016) and Passow (2017).

Most small structural variants overlapping genes were in introns (27,191 variants across 10,196 genes), suggesting intronic variants are common. Among these, 361 genes also fell within highly differentiated regions. Additionally, we identified 4,650 structural variants in putative promoter regions. Of variants in putative promoters, 63 overlapped with highly differentiated regions. In the Puyacatengo drainage, 275 genes were significantly upregulated and 491 downregulated (logFC > 1 or < − 1, BH-adjusted *p*-value < 0.05), while the Tacotalpa drainage showed 774 upregulated and 519 downregulated genes. Of these, 115 upregulated and 152 downregulated genes were shared between drainages. Forty-eight of the shared upregulated and 70 of the shared downregulated genes contain at least one intronic structural variant, with many containing multiple variants and 7 up- and 19 downregulated genes containing promoter variants (Table S6).

To assess whether the overlap between noncoding structural variants and differentially expressed genes exceeded random expectations, we performed a permutation analysis. We found that 119 differentially expressed genes overlapped promoter or intronic structural variants, which was greater than expected by chance (expected mean = 88.3 genes; 95% interval = 74–104; empirical p = 9.999 × 10⁻^5^). However, only 4 of the 292 noncoding structural variants overlapping differentially expressed genes had allele-frequency estimates after genotyping and filtering, and none were highly differentiated between ecotypes. Thus, while noncoding structural variants overlap differentially expressed genes more often than expected by chance, we interpret these as candidate regulatory variants rather than confirmed differentiated variants underlying expression divergence.

### Structural variation and expression of candidate genes encoding sulfide detoxification enzyme ETHE1

**Fig. 3:**
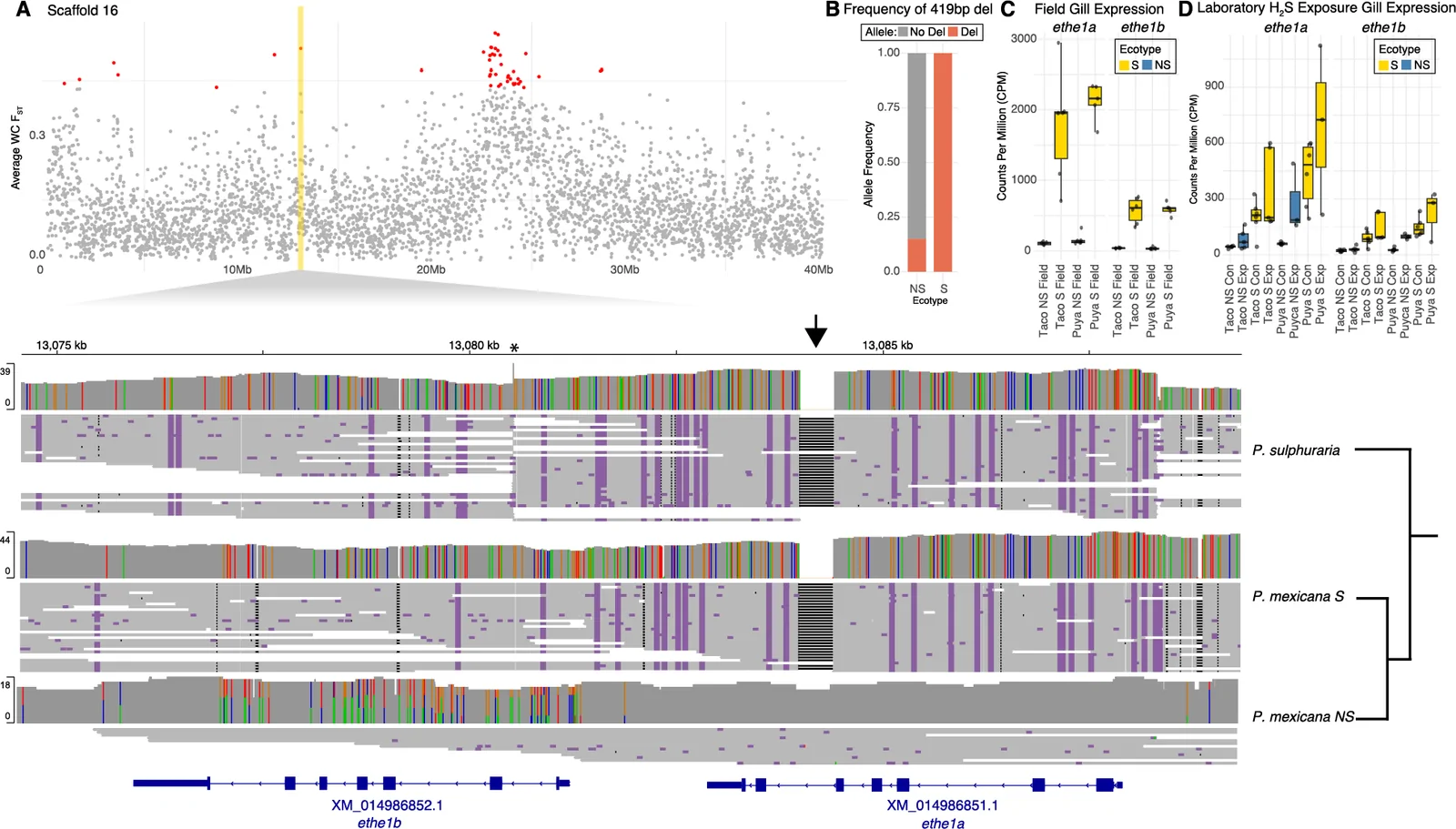
Sequence and gene expression variation in two copies of sulfide processing gene persulfide dioxygenase (*ethe1)*. **A** F_ST_ in 10-kb windows of scaffold 16. Genome wide 99% outlier windows are marked in red. Integrative Genomics Viewer (IGV) screenshot of long reads mapped to the *P. mexicana* NS reference genome and cladogram to show the relationship among populations. The top panel of each row is depth with overlayed colored bars representing SNP locations. In the bottom panel of each row, small insertions are labeled with purple and black lines represent deletions. Structure of *ethe1* copies from genome annotation represented below. A 419 bp deletion shared by both *P. sulphuraria* and P. mexicana S visible in *ethe1a*. A 1.2-kb insertion unique to *P. sulphuraria* in *ethe1b* at 13,080,525 is denoted by a *. **B** Frequency of 419-bp deletion in sulfidic and nonsulfidic individuals. **C** Gene expression in the gill of both copies of *ethe1* in field caught individuals from two population pairs of sulfidic and nonsulfidic populations. Data generated in [33]. **D** Gene expression in the gill of both copies of *ethe1* from individuals exposed to hydrogen sulfide in common garden conditions from two population pairs of sulfidic and nonsulfidic populations. Data originally generated in [45]

We further explored differential expression in both gene copies of ethe1 due to their candidate role in sulfide detoxification, their observed structural variants in promoter and intronic regions, and the highly differentiated variation between ecotypes (Fig. 3A). Gene ethe1a harbored two promoter variants (5,624-bp insertion and 64-bp deletion) and an intronic deletion (419 bp) between exons 6 and 7 that are shared between Taco S and P. sulphuraria and not P. mexicana NS, and a 1.2-kb insertion between exons 1 and 2 unique to P. sulphuraria (Fig. 3A). Of these, the 419-bp deletion is fixed in sulfidic individuals and at low frequency in nonsulfidic individuals (Fig. 3B), with genotypes highly concordant between both breakpoint and coverage based genotyping models. In contrast, structural variation in ethe1b does not sort by ecotype across the genes, as the 1.2-kb insertion is unique to P. sulphuraria.

Expression data from wild-caught gill tissues indicated substantial upregulation of both copies of *ethe1* in sulfide-adapted populations relative to nonsulfidic populations. Specifically, in Tacotalpa, expression was 16-fold higher for *ethe1a* and 14-fold higher for *ethe1b;* similarly, in Puyacatengo, *ethe1a* expression was 13-fold and *ethe1b* was 16-fold higher (Fig. 3C).

Moreover, differential expression between the gene copies is apparent: *ethe1a* exhibited consistently higher expression than *ethe1b* in sulfide-adapted populations (3.1-fold higher in Tacotalpa and 3.6-fold higher in Puyacatengo; Fig. 3C). These expression differences between ecotypes and gene copies, although at a different magnitude, persisted under both common garden nonsulfidic conditions and after experimental H_2_S exposure (Fig. 3D).

To characterize how variation in these genes is distributed among populations, we generated gene trees using data from the 80 individuals analyzed above along with publicly available sequences from Poeciliid fishes (*Gambusia affinis*, *Xiphophorus helleri,* and *Poecilia reticulata*), an outgroup that also shares the ancestral *ethe1* duplication (*Fundulus heteroclitus*), and *Danio rerio*, which contains a single copy *ethe1* (Supplementary Fig. 7). Both gene trees deviate from the established species phylogeny [32, 40, 41]. In both copies of *ethe1* sequences from sulfide adapted *P. mexicana* individuals cluster more closely with *P. sulphuraria* and *P. thermalis* (Pich and Ixta) than with conspecific or sister taxa (Supplementary Fig. 7). But this clustering differs between paralogs: *ethe1a* clusters all sulfidic populations together, with very little variation among individuals, whereas in *ethe1b* only the sulfidic Puya population clusters with *P. sulphuraria* and *P. thermalis* (Pich and Ixta), while the Taco sulfidic population does not.

## Discussion

We explored the role of structural variants in repeated adaptation to hydrogen-sulfide-rich springs in independent lineages of *Poecilia mexicana*. Through an integrative approach combining long-read sequencing, pangenome assembly, and analysis of differentiation with short-reads, we identified 99,016 structural variants across multiple individuals in the *P. mexicana* species complex. Numerous variants were present in regions that were highly differentiated between sulfidic and nonsulfidic ecotypes with some sorting by ecotype and not taxonomic relationship. These variants, distributed across coding, intronic, promoter and intergenic regions, highlight how structural variants could contribute to adaptation, extending beyond the traditionally emphasized single nucleotide polymorphisms (SNPs). But, given these discoveries were made using three genomes across the species complex, they likely underrepresent the full structural variant diversity in the species. For example, recent work on House Finches (*Haemorhous mexicanus*) that utilized a pangenome approach to identify structural variants found over 800,000 structural variants across 16 individuals and an outgroup, spanning ~ 13 million years of evolution across large geographic space [69]. Future work to expand this pangenome to include more individuals as long-read data become available could lead to new insights into independent and shared variation underpinning sulfide tolerance.

### Noncoding structural variants associated with regulatory differences

Structural variants can substantially influence phenotypic evolution without altering protein sequence by affecting regulatory elements and gene expression [11, 70–72]. These regulatory differences may contribute to adaptive divergence under strong environmental selective pressures, such as those found in H_2_S-rich springs. We identified multiple promoters and intronic variants in previously described differentially expressed genes that have been implicated in adaptation [33, 45, 67]. Structural variants in the promoter and intronic regions of *ethe1a* co-occur with highly differentiated single nucleotide polymorphisms between ecotypes and elevated expression of *ethe1a* in sulfidic populations (Fig. 3). These patterns are consistent with a potential role for regulatory variation in shaping expression differences; however, the current data do not allow us to directly infer causal effects of these variants on gene expression and functional validation will be required to establish mechanistic links. Regulatory neofunctionalization has been proposed as a major source of evolutionary innovation [73, 74], but whether such processes are occurring in this system remains to be determined.

In particular, the 419-bp deletion in an intron of *ethe1a* near the intron–exon boundary could influence mRNA processing or stability, although this remains a hypothesis. The presence of similar structural variants in both *P. sulphuraria* and H_2_S -adapted *P. mexicana* (Taco S), but not in closely related nonsulfidic *P. mexicana* populations (Fig. 3A), is consistent with a shared origin, potentially via gene flow or incomplete lineage sorting, although alternative explanations such as parallel evolution cannot be excluded. Future studies comparing exon usage between ecotypes, potentially using long-read RNA-seq, could clarify how these paralogs evolved and how these variants influence expression and splicing of this H₂S-detoxification gene.

### Structural variants affecting transporter and sulfide metabolism genes

We identified variants in transporter and sulfur-processing genes (Figs. 1 & 2), including a highly differentiated large genomic inversion involving the sodium-sulfate cotransporter gene *slc13a1.* Given growing evidence that inversions can maintain adaptive variation and drive ecological divergence [75, 76], this inversion could contribute to the maintenance of linked adaptive variation in the face of low levels of gene flow occurring between ecotypes in at least some populations [38, 68]. Additional variants exist in these and related transport genes, including a 195-bp insertion within exon 1 of sulfate transporter *slc26a*2, a 60-bp deletion in exon 7 of *slc13a1*, and a 143-bp insertion in carbohydrate sulfotransferase 6 (*chst*). Future studies using rapidly advancing protein prediction models [77] could shed light on the functional impacts of these, and other, coding variants in highly differentiated regions. Moreover, GO enrichment analysis revealed significant overrepresentation of many transport-related biological processes among genes harboring structural variants within highly differentiated genomic regions (Fig. 2B). These results are consistent with a complex genomic response encompassing both regulatory and protein-coding modifications to environmental H_2_S exposure.

The presence of variants in both sulfur processing genes and transporter genes leads to new hypotheses about the mechanisms underlying repeated adaptation to sulfide springs. H_2_S is produced endogenously in the body at low levels and is important in a variety of biological processes, ranging from cell signaling to proper neurological and cardiac function [78–82]. There are a variety of conserved mechanisms for processing surplus H_2_S and related compounds to maintain tight regulation, given its inhibition of cytochrome c oxidase and mitochondrial respiration even at low concentrations [83–85]. These H_2_S regulatory mechanisms, via sulfide-quinone oxidoreductase (*sqor*) [86] or superoxide dismutase [Cu–Zn] (*sod1*) [87] in the mitochondria, produce a variety of downstream byproducts, such as sulfate and thiosulfate. Given evidence for differential SQR activity between ecotypes [32] as well as differential expression of multiple sulfur processing genes between ecotypes [32, 33, 45, 67, 68], it is likely that in addition to oxidizing H_2_S to prevent the disruption of the electrochemical gradient, cells need to excrete the byproducts of this process to maintain homeostasis. Given that these byproducts need transporters to move across membranes, the enrichment in variants associated with sulfate transport is consistent with the possibility that modification of excretory pathways complements H_2_S oxidization in mediating elevated tolerance to environmental H_2_S. The presence of ecotype-specific structural variants occurring in transport genes parallels previous findings, where *slc26a5* was duplicated and upregulated in sulfidic ecotypes [41]. In our dataset, we identified a nearby structural variant annotated as an ~ 8.1 kb insertion; however, it is unclear whether this corresponds to the previously reported duplication because the variant did not map to *slc26a5* based on sequence similarity. Brown [41] also found a duplication in *slc13a1*, but they did not find the smaller deletion within the gene identified in this study. In our dataset, we did not detect a duplication similar to that identified in Brown [41]. However, two annotated copies of this gene occur within the large inversion on scaffold 6, suggesting that duplication at this locus may be associated with the inversion rather than a separate duplication event. Future physiological research assessing not only the function of detoxification related proteins, but also of transport of byproducts across the membrane could shed light into the complexity of adaptation to sulfide springs.

### Broader evolutionary significance of structural variation

Identifying genetic variants underpinning adaptive phenotypes remains challenging due to complex genotype-to-phenotype relationships. Nonetheless, our findings clearly illustrate that structural variants are present, and often common, in genomic regions putatively under selection [40]. These variants could play an integral role in repeated adaptation to H_2_S-rich springs. Our results complement prior studies highlighting adaptive divergence in gene expression and physiological H_2_S tolerance between ecotypes [32–34, 67, 68, 88], suggesting structural variants may act alongside SNP-driven mechanisms by modulating gene expression, splicing, and/or protein function.

Importantly, the repeated identification of identical structural variants across independently evolved sulfidic populations raises intriguing questions regarding their evolutionary origin. Given the improbability of identical mutations independently arising multiple times, our results are consistent with the persistence of ancestral standing genetic variation or adaptive introgression mediated through gene flow among closely related lineages, suggested but not rigorously tested across populations in previous studies [32, 40, 68]. Future population genomic studies will be essential for distinguishing these scenarios, further elucidating evolutionary processes underlying adaptive genomic variation.

## Conclusion

This study provides evidence that structural variants could contribute to the repeated ecological adaptation, emphasizing the importance of integrating structural variants into future studies of local adaptation. These new insights pave the way to novel investigations into how structural variants influence both protein structure and gene regulation and drive adaptive divergence across natural populations.

## Supplementary Information

Supplementary Material 1.

Supplementary Material 2.

Supplementary Material 3.

Supplementary Material 4.

## Data Availability

The dataset supporting the conclusions of this article is available in the NCBI repository, BioProject PRJNA1128810 and https://www.ncbi.nlm.nih.gov/bioproject/?term=PRJNA1128810. All short-read sequencing data is available on NCBI with the SRR accession numbers provided in Table S3. All code used for the analysis and visualization of these data, as well as individual and genus-specific VCFs, can be found at https://github.com/kara-ry/Poeciliid_H2S_SVs.

## References

[1] Rappaport HB, Oliverio AM. Lessons from extremophiles: functional adaptations and genomic innovations across the eukaryotic tree of life. Genome Biol Evol. 2024;16:evae160. 10.1093/gbe/evae160 PMC11299111 39101574

[2] Balart-García P, Aristide L, Bradford TM, Beasley-Hall PG, Polak S, Cooper SJB, et al. Parallel and convergent genomic changes underlie independent subterranean colonization across beetles. Nat Commun. 2023;14:3842. 10.1038/s41467-023-39603-1 PMC10310748 37386018

[3] Culver DC, Pipan T. The biology of caves and other subterranean habitats. Oxford, England: Oxford University Press; 2009.

[4] Christiansen K. Convergence and parallelism in cave Entomobryinae. Evolution. 1961;15:288.

[5] Derkarabetian S, Steinmann DB, Hedin M. Repeated and time-correlated morphological convergence in cave-dwelling harvestmen (Opiliones, Laniatores) from Montane Western North America. PLoS ONE. 2010;5:e10388. 10.1371/journal.pone.0010388 PMC2866537 20479884

[6] Liu Z, Qi F-Y, Zhou X, Ren H-Q, Shi P. Parallel sites implicate functional convergence of the hearing gene Prestin among echolocating mammals. Mol Biol Evol. 2014;31:2415–24. 10.1093/molbev/msu194 24951728

[7] Corbett-Detig RB, Russell SL, Nielsen R, Losos J. Phenotypic convergence is not mirrored at the protein level in a lizard adaptive radiation. Mol Biol Evol. 2020;37:1604–14. 10.1093/molbev/msaa028 32027369

[8] Morales AE, Burbrink FT, Segall M, Meza M, Munegowda C, Webala PW, et al. Distinct genes with similar functions underlie convergent evolution in *Myotis* bat ecomorphs. Mol Biol Evol. 2024;41:msae165. 10.1093/molbev/msae165 PMC11371419 39116340

[9] Xu S, Wang J, Guo Z, He Z, Shi S. Genomic convergence in the adaptation to extreme environments. Plant Commun. 2020;1:100117. 10.1016/j.xplc.2020.100117 PMC7747959 33367270

[10] Li F, Qiao Z, Duan Q, Nevo E. Adaptation of mammals to hypoxia. Anim Models Exp Med. 2021;4:311–8. 10.1002/ame2.12189 PMC8690989 34977482

[11] Scott AJ, Chiang C, Hall IM. Structural variants are a major source of gene expression differences in humans and often affect multiple nearby genes. Genome Res. 2021;31:2249–57. 10.1101/gr.275488.121 PMC8647827 34544830

[12] Lin M, Whitmire S, Chen J, Farrel A, Shi X, Guo J-T. Effects of short indels on protein structure and function in human genomes. Sci Rep. 2017;7:9313. 10.1038/s41598-017-09287-x PMC5570956 28839204

[13] Jilani M, Turcan A, Haspel N, Jagodzinski F. Elucidating the structural impacts of protein InDels. Biomolecules. 2022;12:1435. 10.3390/biom12101435 PMC9599607 36291643

[14] McClintock B. Cytological observations of deficiencies involving known genes, translocations and an inversion in Zea mays. Research Bulletin. Missouri Agricultural Experiment Station, Vol. 163, 1931. p. 30.

[15] Sturtevant AH. The linear arrangement of six sex-linked factors in *Drosophila*, as shown by their mode of association. J Exp Zool. 1913;14:43–59.

[16] Berdan EL, Aubier TG, Cozzolino S, Faria R, Feder JL, Giménez MD, et al. Structural variants and speciation: multiple processes at play. Cold Spring Harb Perspect Biol. 2024;16:a041446. 10.1101/cshperspect.a041446 PMC10910405 38052499

[17] Mérot C, Oomen RA, Tigano A, Wellenreuther M. A roadmap for understanding the evolutionary significance of structural genomic variation. Trends Ecol Evol. 2020;35:561–72. 10.1016/j.tree.2020.03.002 32521241

[18] Zhang L, Reifová R, Halenková Z, Gompert Z. How important are structural variants for speciation? Genes. 2021;12:1084. 10.3390/genes12071084 PMC8305853 34356100

[19] Joron M, Frezal L, Jones RT, Chamberlain NL, Lee SF, Haag CR, et al. Chromosomal rearrangements maintain a polymorphic supergene controlling butterfly mimicry. Nature. 2011;477:203–6. 10.1038/nature10341 PMC3717454 21841803

[20] Kim K-W, De-Kayne R, Gordon IJ, Omufwoko KS, Martins DJ, ffrench-Constant R, et al. Stepwise evolution of a butterfly supergene via duplication and inversion. Philos Trans R Soc Lond B Biol Sci. 2022;377:20210207. 10.1098/rstb.2021.0207 PMC9189502 35694743

[21] Mérot C, Llaurens V, Normandeau E, Bernatchez L, Wellenreuther M. Balancing selection via life-history trade-offs maintains an inversion polymorphism in a seaweed fly. Nat Commun. 2020;11:670. 10.1038/s41467-020-14479-7 PMC6997199 32015341

[22] Küpper C, Stocks M, Risse JE, dos Remedios N, Farrell LL, McRae SB, et al. A supergene determines highly divergent male reproductive morphs in the ruff. Nat Genet. 2016;48:79–83. 10.1038/ng.3443 PMC5218575 26569125

[23] Yan Z, Martin SH, Gotzek D, Arsenault SV, Duchen P, Helleu Q, et al. Evolution of a supergene that regulates a trans-species social polymorphism. Nat Ecol Evol. 2020;4:240–9. 10.1038/s41559-019-1081-1 31959939

[24] Ishikawa A, Stuart YE, Bolnick DI, Kitano J. Copy number variation of a fatty acid desaturase gene *Fads2* associated with ecological divergence in freshwater stickleback populations. Biol Lett. 2021;17:20210204. 10.1098/rsbl.2021.0204 PMC8385377 34428959

[25] Kirubakaran TG, Grove H, Kent MP, Sandve SR, Baranski M, Nome T, et al. Two adjacent inversions maintain genomic differentiation between migratory and stationary ecotypes of Atlantic cod. Mol Ecol. 2016;25:2130–43. 10.1111/mec.13592 26923504

[26] Dulai KS, von Dornum M, Mollon JD, Hunt DM. The evolution of trichromatic color vision by opsin gene duplication in New World and Old World primates. Genome Res. 1999;9:629–38. 10413401

[27] Frentiu FD, Bernard GD, Sison-Mangus MP, Brower AVZ, Briscoe AD. Gene duplication is an evolutionary mechanism for expanding spectral diversity in the long-wavelength photopigments of butterflies. Mol Biol Evol. 2007;24:2016–28. 10.1093/molbev/msm132 17609538

[28] Bertolotti AC, Layer RM, Gundappa MK, Gallagher MD, Pehlivanoglu E, Nome T, et al. The structural variation landscape in 492 Atlantic salmon genomes. Nat Commun. 2020;11:5176. 10.1038/s41467-020-18972-x PMC7560756 33056985

[29] Mérot C, Stenløkk KSR, Venney C, Laporte M, Moser M, Normandeau E, et al. Genome assembly, structural variants, and genetic differentiation between lake whitefish young species pairs (Coregonus sp.) with long and short reads. Mol Ecol. 2023;32:1458–77. 10.1111/mec.16468 35416336

[30] Stuart KC, Edwards RJ, Sherwin WB, Rollins LA. Contrasting patterns of single nucleotide polymorphisms and structural variation across multiple invasions. Mol Biol Evol. 2023;40:msad046. 10.1093/molbev/msad046 PMC10037079 36814414

[31] Feulner PGD, De‐Kayne R. Genome evolution, structural rearrangements and speciation. J Evol Biol. 2017;30:1488–90. 10.1111/jeb.13101 28786195

[32] Greenway R, Barts N, Henpita C, Brown AP, Rodriguez LA, Peña CMR, et al. Convergent evolution of conserved mitochondrial pathways underlies repeated adaptation to extreme environments. Proc Natl Acad Sci U S A. 2020;117:16424–30. 10.1073/pnas.2004223117 PMC7368198 32586956

[33] Kelley JL, Arias-Rodriguez L, Patacsil Martin D, Yee M-C, Bustamante CD, Tobler M. Mechanisms Underlying Adaptation to Life in Hydrogen Sulfide-Rich Environments. Mol Biol Evol. 2016;33:1419–34. 10.1093/molbev/msw020 PMC4868117 26861137

[34] Tobler M, Passow CN, Greenway R, Kelley JL, Shaw JH. The evolutionary ecology of animals inhabiting hydrogen sulfide–rich environments. Annu Rev Ecol Evol Syst. 2016;47:239–62.

[35] Tobler M, Kelley JL, Plath M, Riesch R. Extreme environments and the origins of biodiversity: adaptation and speciation in sulphide spring fishes. Mol Ecol. 2018;27:843–59. 10.1111/mec.14497 29368386

[36] Plath M, Pfenninger M, Lerp H, Riesch R, Eschenbrenner C, Slattery PA, et al. Genetic differentiation and selection against migrants in evolutionarily replicated extreme environments. Evolution. 2013;67:2647–61. 10.1111/evo.12133 24033173

[37] Riesch R, Plath M, Schlupp I, Tobler M, Langerhans RB. Colonisation of toxic environments drives predictable life-history evolution in livebearing fishes (Poeciliidae). Ecol Lett. 2014;17:65–71. 10.1111/ele.12209 24188245

[38] Greenway R, De-Kayne R, Brown AP, Camarillo H, Delich C, McGowan KL, et al. Integrative analyses of convergent adaptation in sympatric extremophile fishes. Curr Biol. 2024. 10.1016/j.cub.2024.09.027. 39395416

[39] Pfenninger M, Lerp H, Tobler M, Passow C, Kelley JL, Funke E, et al. Parallel evolution of cox genes in H2S-tolerant fish as key adaptation to a toxic environment. Nat Commun. 2014;5:3873. 10.1038/ncomms4873 24815812

[40] Ryan K, Greenway R, Landers J, Arias-Rodriguez L, Tobler M, Kelley JL. Selection on standing genetic variation mediates convergent evolution in extremophile fish. Mol Ecol. 2023;32:5042–54. 10.1111/mec.17081 37548336

[41] Brown AP, McGowan KL, Schwarzkopf EJ, Greenway R, Rodriguez LA, Tobler M, et al. Local ancestry analysis reveals genomic convergence in extremophile fishes. Philos Trans R Soc Lond B Biol Sci. 2019;374:20180240. 10.1098/rstb.2018.0240 PMC6560267 31154969

[42] Ryan K, De-Kayne R, Davis J, Arias-Rodriguez L, Tobler M, Kelley JL. New genome assemblies for Poeciliidae: a foundation for adaptation studies. Genome Biol Evol. 2025;17:evaf111. 10.1093/gbe/evaf111 PMC12204701 40548548

[43] Mahmoud M, Gobet N, Cruz-Dávalos DI, Mounier N, Dessimoz C, Sedlazeck FJ. Structural variant calling: the long and the short of it. Genome Biol. 2019;20:246. 10.1186/s13059-019-1828-7 PMC6868818 31747936

[44] Begum G, Albanna A, Bankapur A, Nassir N, Tambi R, Berdiev BK, et al. Long-read sequencing improves the detection of structural variations impacting complex non-coding elements of the genome. Int J Mol Sci. 2021;22:2060. 10.3390/ijms22042060 PMC7923155 33669700

[45] Passow CN, Henpita C, Shaw JH, Quackenbush CR, Warren WC, Schartl M, et al. The roles of plasticity and evolutionary change in shaping gene expression variation in natural populations of extremophile fish. Mol Ecol. 2017;26:6384–99. 10.1111/mec.14360 28926156

[46] Li H. Minimap2: pairwise alignment for nucleotide sequences. Bioinformatics. 2018;34:3094–100. 10.1093/bioinformatics/bty191 PMC6137996 29750242

[47] Saunders CT, Holt JM, Baker DN, Lake JA, Belyeu JR, Kronenberg Z, et al. Sawfish: improving long-read structural variant discovery and genotyping with local haplotype modeling. Bioinformatics. 2025;41:btaf136. 10.1093/bioinformatics/btaf136 PMC12000528 40203061

[48] Smolka M, Paulin LF, Grochowski CM, Horner DW, Mahmoud M, Behera S, et al. Detection of mosaic and population-level structural variants with Sniffles2. Nat Biotechnol. 2024;42:1571–80. 10.1038/s41587-023-02024-y PMC11217151 38168980

[49] Danecek P, Bonfield JK, Liddle J, Marshall J, Ohan V, Pollard MO, et al. Twelve years of SAMtools and BCFtools. Gigascience. 2021;10:giab008. 10.1093/gigascience/giab008 PMC7931819 33590861

[50] Cheng H, Concepcion GT, Feng X, Zhang H, Li H. Haplotype-resolved de novo assembly using phased assembly graphs with hifiasm. Nat Methods. 2021;18:170–5. 10.1038/s41592-020-01056-5 PMC7961889 33526886

[51] Hickey G, Monlong J, Ebler J, Novak AM, Eizenga JM, Gao Y, et al. Pangenome graph construction from genome alignments with Minigraph-Cactus. Nat Biotechnol. 2024;42:663–73. 10.1038/s41587-023-01793-w PMC10638906 37165083

[52] Li H, Feng X, Chu C. The design and construction of reference pangenome graphs with minigraph. Genome Biol. 2020;21:265. 10.1186/s13059-020-02168-z PMC7568353 33066802

[53] Garrison E, Sirén J, Novak AM, Hickey G, Eizenga JM, Dawson ET, et al. Variation graph toolkit improves read mapping by representing genetic variation in the reference. Nat Biotechnol. 2018;36:875–9. 10.1038/nbt.4227 PMC6126949 30125266

[54] Garrison, E. vcfbub: use variant nesting information to flter overlapping sites from vg deconstruct output. Zenodo. https://zenodo.org/records/7239225.

[55] English AC, Menon VK, Gibbs RA, Metcalf GA, Sedlazeck FJ. Truvari: refined structural variant comparison preserves allelic diversity. Genome Biol. 2022;23:271. 10.1186/s13059-022-02840-6 PMC9793516 36575487

[56] Kirsche M, Prabhu G, Sherman R, Ni B, Battle A, Aganezov S, et al. Jasmine and Iris: population-scale structural variant comparison and analysis. Nat Methods. 2023;20:408–17. 10.1038/s41592-022-01753-3 PMC10006329 36658279

[57] Jeffares DC, Jolly C, Hoti M, Speed D, Shaw L, Rallis C, et al. Transient structural variations have strong effects on quantitative traits and reproductive isolation in fission yeast. Nat Commun. 2017;8:14061. 10.1038/ncomms14061 PMC5286201 28117401

[58] Andrews S. FastQC A Quality Control tool for High Throughput Sequence Data. 2010. https://www.bioinformatics.babraham.ac.uk/projects/fastqc/.

[59] Chen S, Zhou Y, Chen Y, Gu J. Fastp: an ultra-fast all-in-one FASTQ preprocessor. Bioinformatics. 2018;34:i884–90. 10.1093/bioinformatics/bty560 PMC6129281 30423086

[60] Vasimuddin M, Misra S, Li H, Aluru S. Efficient architecture-aware acceleration of BWA-MEM for multicore systems. In: 2019 IEEE International Parallel and Distributed Processing Symposium (IPDPS). IEEE; 2019. 10.1109/ipdps.2019.00041.

[61] Tarasov A, Vilella AJ, Cuppen E, Nijman IJ, Prins P. Sambamba: fast processing of NGS alignment formats. Bioinformatics. 2015;31:2032–4. 10.1093/bioinformatics/btv098 PMC4765878 25697820

[62] Danecek P, Auton A, Abecasis G, Albers CA, Banks E, DePristo MA, et al. The variant call format and VCFtools. Bioinformatics. 2011;27:2156–8. 10.1093/bioinformatics/btr330 PMC3137218 21653522

[63] Korunes KL, Samuk K. Pixy: unbiased estimation of nucleotide diversity and divergence in the presence of missing data. Mol Ecol Resour. 2021;21:1359–68. 10.1111/1755-0998.13326 PMC8044049 33453139

[64] Weir BS, Cockerham CC. Estimating F-statistics for the analysis of population structure. Evolution. 1984;38:1358–70. 10.1111/j.1558-5646.1984.tb05657.x 28563791

[65] Eggertsson HP, Kristmundsdottir S, Beyter D, Jonsson H, Skuladottir A, Hardarson MT, et al. GraphTyper2 enables population-scale genotyping of structural variation using pangenome graphs. Nat Commun. 2019;10:5402. 10.1038/s41467-019-13341-9 PMC6881350 31776332

[66] Sedlazeck FJ, Rescheneder P, Smolka M, Fang H, Nattestad M, von Haeseler A, et al. Accurate detection of complex structural variations using single-molecule sequencing. Nat Methods. 2018;15:461–8. 10.1038/s41592-018-0001-7 PMC5990442 29713083

[67] Passow CN, Brown AP, Arias-Rodriguez L, Yee M-C, Sockell A, Schartl M, et al. Complexities of gene expression patterns in natural populations of an extremophile fish (*Poecilia mexicana*, Poeciliidae). Mol Ecol. 2017;26:4211–25. 10.1111/mec.14198 PMC5731456 28598519

[68] Brown AP, Arias-Rodriguez L, Yee M-C, Tobler M, Kelley JL. Concordant changes in gene expression and nucleotides underlie independent adaptation to hydrogen-sulfide-rich environments. Genome Biol Evol. 2018;10:2867–81. 10.1093/gbe/evy198 PMC6225894 30215710

[69] Fang B, Edwards SV. Fitness consequences of structural variation inferred from a House Finch pangenome. Proc Natl Acad Sci U S A. 2024;121:e2409943121. 10.1073/pnas.2409943121 PMC11588099 39531493

[70] Shaul O. How introns enhance gene expression. Int J Biochem Cell Biol. 2017;91 Pt B:145–55. 10.1016/j.biocel.2017.06.016 28673892

[71] Harewood L, Chaignat E, Reymond A. Structural variation and its effect on expression. Methods Mol Biol. 2012;838:173–86. 10.1007/978-1-61779-507-7_8 22228012

[72] Chaisson MJP, Wilson RK, Eichler EE. Genetic variation and the de novo assembly of human genomes. Nat Rev Genet. 2015;16:627–40. 10.1038/nrg3933 PMC4745987 26442640

[73] Moore RC, Purugganan MD. The evolutionary dynamics of plant duplicate genes. Curr Opin Plant Biol. 2005;8:122–8. 10.1016/j.pbi.2004.12.001 15752990

[74] Ohno S. Evolution by gene duplication. Berlin, Germany: Springer; 1970. 10.1007/978-3-642-86659-3.

[75] Thorstensen MJ, Euclide PT, Jeffrey JD, Shi Y, Treberg JR, Watkinson DA, et al. A chromosomal inversion may facilitate adaptation despite periodic gene flow in a freshwater fish. Ecol Evol. 2022;12:e8898. 10.1002/ece3.8898 PMC9077824 35571758

[76] Zong S, Li Y-L, Liu J-X. Genomic architecture of rapid parallel adaptation to fresh water in a wild fish. Mol Biol Evol. 2020;38:1317–29. 10.1093/molbev/msaa290 PMC8480189 33146383

[77] Jumper J, Evans R, Pritzel A, Green T, Figurnov M, Ronneberger O, et al. Highly accurate protein structure prediction with AlphaFold. Nature. 2021;596:583–9. 10.1038/s41586-021-03819-2 PMC8371605 34265844

[78] Cao X, Ding L, Xie Z-Z, Yang Y, Whiteman M, Moore PK, et al. A review of hydrogen sulfide synthesis, metabolism, and measurement: is modulation of hydrogen sulfide a novel therapeutic for cancer? Antioxid Redox Signal. 2019;31:1–38. 10.1089/ars.2017.7058 PMC6551999 29790379

[79] García-Bereguiaín MA, Samhan-Arias AK, Martín-Romero FJ, Gutiérrez-Merino C. Hydrogen sulfide raises cytosolic calcium in neurons through activation of L-type Ca2+ channels. Antioxid Redox Signal. 2008;10:31–42. 10.1089/ars.2007.1656 17956188

[80] Kabil O, Motl N, Banerjee R. H2S and its role in redox signaling. Biochimica et Biophysica Acta (BBA) - Proteins and Proteomics. 2014;1844:1355–66. 10.1016/j.bbapap.2014.01.002 PMC4048824 24418393

[81] Peers C, Bauer CC, Boyle JP, Scragg JL, Dallas ML. Modulation of ion channels by hydrogen sulfide. Antioxid Redox Signal. 2012;17:95–105. 10.1089/ars.2011.4359 22074224

[82] Li L, Rose P, Moore PK. Hydrogen sulfide and cell signaling. Annu Rev Pharmacol Toxicol. 2011;51:169–87. 10.1146/annurev-pharmtox-010510-100505 21210746

[83] Jiang J, Chan A, Ali S, Saha A, Haushalter KJ, Lam W-LM, et al. Hydrogen sulfide—mechanisms of toxicity and development of an antidote. Sci Rep. 2016;6:20831. 10.1038/srep20831 PMC4753484 26877209

[84] Cooper CE, Brown GC. The inhibition of mitochondrial cytochrome oxidase by the gases carbon monoxide, nitric oxide, hydrogen cyanide and hydrogen sulfide: chemical mechanism and physiological significance. J Bioenerg Biomembr. 2008;40:533–9. 10.1007/s10863-008-9166-6 18839291

[85] Szabo C. A timeline of hydrogen sulfide (H2S) research: from environmental toxin to biological mediator. Biochem Pharmacol. 2018;149:5–19. 10.1016/j.bcp.2017.09.010 PMC5862769 28947277

[86] Landry AP, Ballou DP, Banerjee R. H2S oxidation by nanodisc-embedded human sulfide quinone oxidoreductase. J Biol Chem. 2017;292:11641–9. 10.1074/jbc.M117.788547 PMC5512061 28512131

[87] Switzer CH, Kasamatsu S, Ihara H, Eaton P. SOD1 is an essential H2S detoxifying enzyme. Proc Natl Acad Sci U S A. 2023;120:e2205044120. 10.1073/pnas.2205044120 PMC9934061 36630448

[88] Perry BW, McGowan KL, Arias-Rodriguez L, Duttke SH, Tobler M, Kelley JL. Nascent transcription reveals regulatory changes in extremophile fishes inhabiting hydrogen sulfide-rich environments. Proc Biol Sci. 2024;291:20240412. 10.1098/rspb.2024.0412 PMC11285508 38889788

[89] Robinson MD, McCarthy DJ, Smyth GK. edgeR: a Bioconductor package for differential expression analysis of digital gene expression data. Bioinformatics. 2010;26(1):139–40. 10.1093/bioinformatics/btp616. Epub 2009 Nov 11. PMID: 19910308; PMCID: PMC2796818. PMC2796818 19910308

[90] Katoh K, Standley DM. MAFFT multiple sequence alignment software version 7: improvements in performance and usability. Mol Biol Evol. 2013;30(4):772–80. 10.1093/molbev/mst010. Epub 2013 Jan 16. PMID: 23329690; PMCID: PMC3603318. PMC3603318 23329690

[91] Nguyen LT, Schmidt HA, von Haeseler A, Minh BQ. IQ-TREE: a fast and effective stochastic algorithm for estimating maximum-likelihood phylogenies. Mol Biol Evol. 2015;32(1):268–74. 10.1093/molbev/msu300. Epub 2014 Nov 3. PMID: 25371430; PMCID: PMC4271533. PMC4271533 25371430

