# Supplementary material for: Ecotype-associated structural variants across multiple locally adapted Poeciliid fishes
Source: BMC Genomics. 2026 Aug 1;27:898. doi: 10.1186/s12864-026-13205-9 (PMC13721252; doi:10.1186/s12864-026-13205-9)
Supplement: Supplementary file 1 [file 12864_2026_13205_MOESM1_ESM.pdf]

Supplemental Information for:

Pangenome-based analysis reveals ecotype-associated structural variants across multiple locally adapted Poeciliid fishes

Kara Ryan, Rishi De-Kayne, Lenin Arias-Rodriguez, Michael Tobler, Joanna L. Kelley

Table S1: Focal populations included in this study including the species name, site name, site ID used in the manuscript, the drainage of origin, the habitat type, either sulfidic (S) or nonsulfidic (NS) springs, latitude and longitude of the population, the coverage for samples with PacBio HiFi reads and the number of individuals per population sequenced with Illumina sequencing in this study.

Species	Site name	Site ID	Drainage	Habitat	Lat & Lon	Estimated HiFi coverage	Median HiFi read length (bp)	Illumina sample size
P. sulphuraria	Baños del Azufre	Pich 1 S	Pichucalco	S	17.552, -92.998			8
P. mexicana	Vet Station	Pich 1 NS	Pichucalco	NS	17.556, -93.008			8
P. sulphuraria	La Gloria	Pich 2 S	Pichucalco	S	17.532, -93.015	32X	10,433	8
P. mexicana	Rosita	Pich 2 NS	Pichucalco	NS	17.485 -93.103			8
P. thermalis	La Esperanza	Ixta S	Ixtapangajoya	S	17.511, -92.980			8
P. mexicana	Rio Ixtapangajoya	Ixta NS	Ixtapangajoya	NS	17.495, -92.998			8
P. mexicana	La Lluvia, small spring	Puya S	Puyacatengo	S	17.463, -92.895			8
P. mexicana	Vicente Guerrero	Puya NS	Puyacatengo	NS	17.510, -92.914			8
P. mexicana	El Azufre I	Taco S	Tacotalpa	S	17.442, -92.774	34X	10,263	8
P. mexicana	Bonita	Taco NS	Tacotalpa	NS	17.426, -92.752	22X	11,005	8

Table S2: Haplotype assemblies generated using HiFiasm including the species, site ID, habitat type, number of contigs assembled and the N50 (bp) for each haplotype assembly.

Species	Site ID	Habitat	Hap 1 contigs	Hap 1 N50	Hap 2 contigs	Hap 2 N50
P. mexicana	Taco NS	Nonsulfidic	405	5,429,777	315	7,850,947
P. mexicana	Taco S	Sulfidic	164	27,106,189	169	26,192,946
P. sulphuraria	Pich2 S	Sulfidic	161	23,574,294	113	25,996,582

Table S3: Sample IDs from BioProject PRJNA1128810 used in this study, including estimated sequence coverage. Provided as separate Excel file "Pmex_SV_table_S3"

Table S4: Allele frequencies of structural variants and gene annotation if it occurs in a gene. Provided as a separate Excel file "Pmex_SV_table_S4". Gene IDs may appear multiple times because multiple structural variants with distinct coordinates can overlap the same annotated gene. Each row represents a separate variant call.

Table S5: Table of genes present in F_{ST} outlier windows. Provided as separate Excel file "Pmex_SV_table_S5"

Table S6: Allele frequency difference between all genotyped variants. Provided as separate Excel file "Pmex_SV_table_S6"

Table S7: Shared, upregulated and downregulated genes containing structural variants in intronic or promoter regions.

**Gene ID**	**Gene name**	**Differential expression**
ethe1a	Persulfide dioxygenase	Upregulated
ppargc1b	peroxisome proliferator-activated receptor gamma coactivator 1-beta	Upregulated
gsr	glutathione reductase	Upregulated
pxdn	peroxidasin homolog	Upregulated
cdip1	cell death-inducing p53-target protein 1 homolog	Upregulated
apol6	apolipoprotein L6-like	Upregulated
LOC106913869	long noncoding RNA	Upregulated
slc4a4	Electrogenic sodium bicarbonate cotransporter 1	Downregulated
atp5j	ATP synthase-coupling factor 6	Downregulated
abo	histo-blood group ABO system transferase	Downregulated
map3k5	Mitogen-activated protein kinase 5	Downregulated
cish	Cytokine-inducible SH2-containing protein	Downregulated
krt16	Keratin, type I cytoskeletal 16	Downregulated
cyp3a5	Cytochrome P450 3A5	Downregulated
ccamc2	Calcium-binding mitochondrial carrier protein	Downregulated
clc9a7	sodium/hydrogen exchanger 7	Downregulated

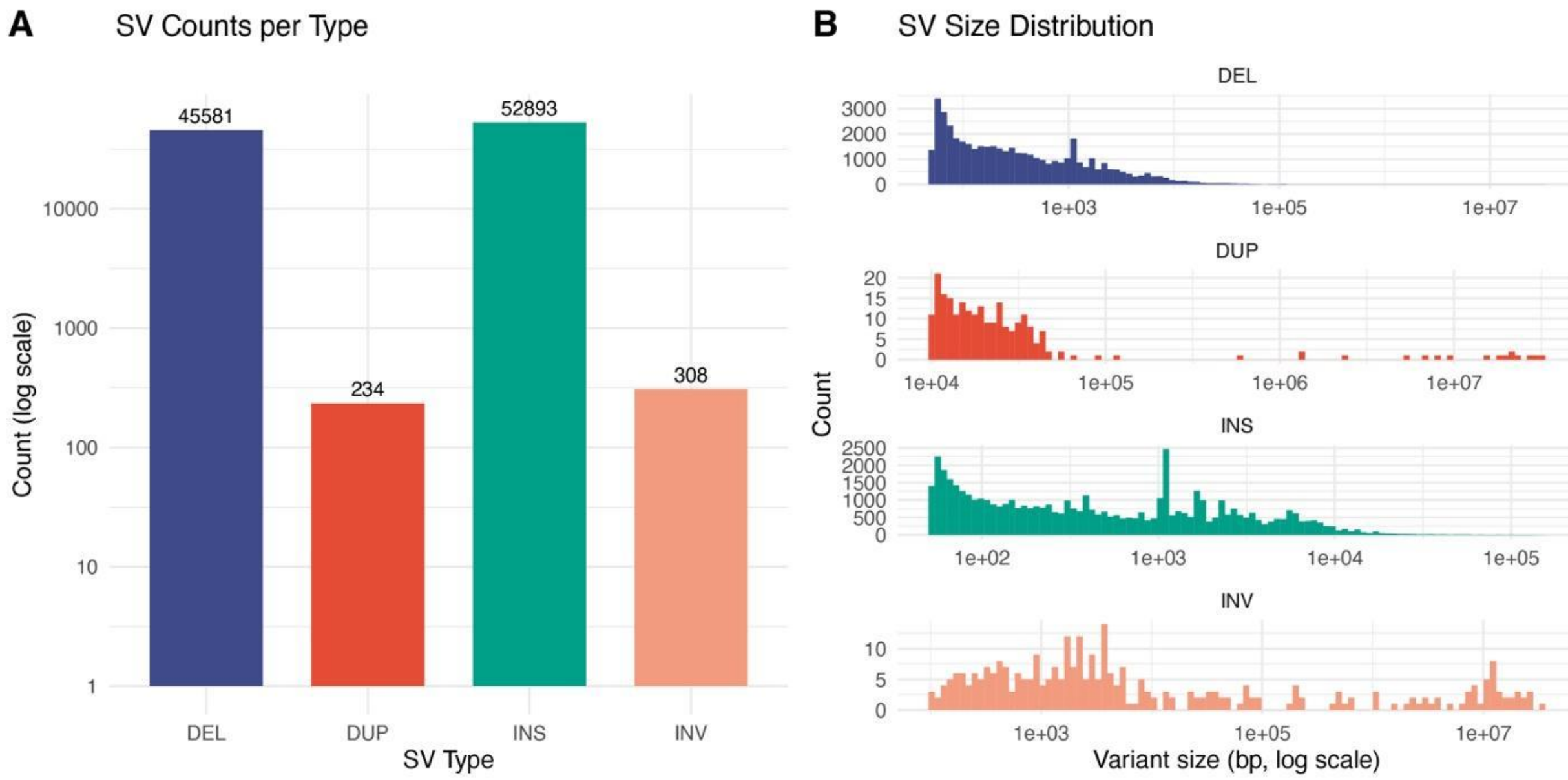

Figure S1: (A) Count of variants found after merging via three methods of variant discovery for each variant type, including deletions (DEL), duplications (DUP), insertions (INS), and inversions (INV). (B) The size distribution of variants found for each variant type.

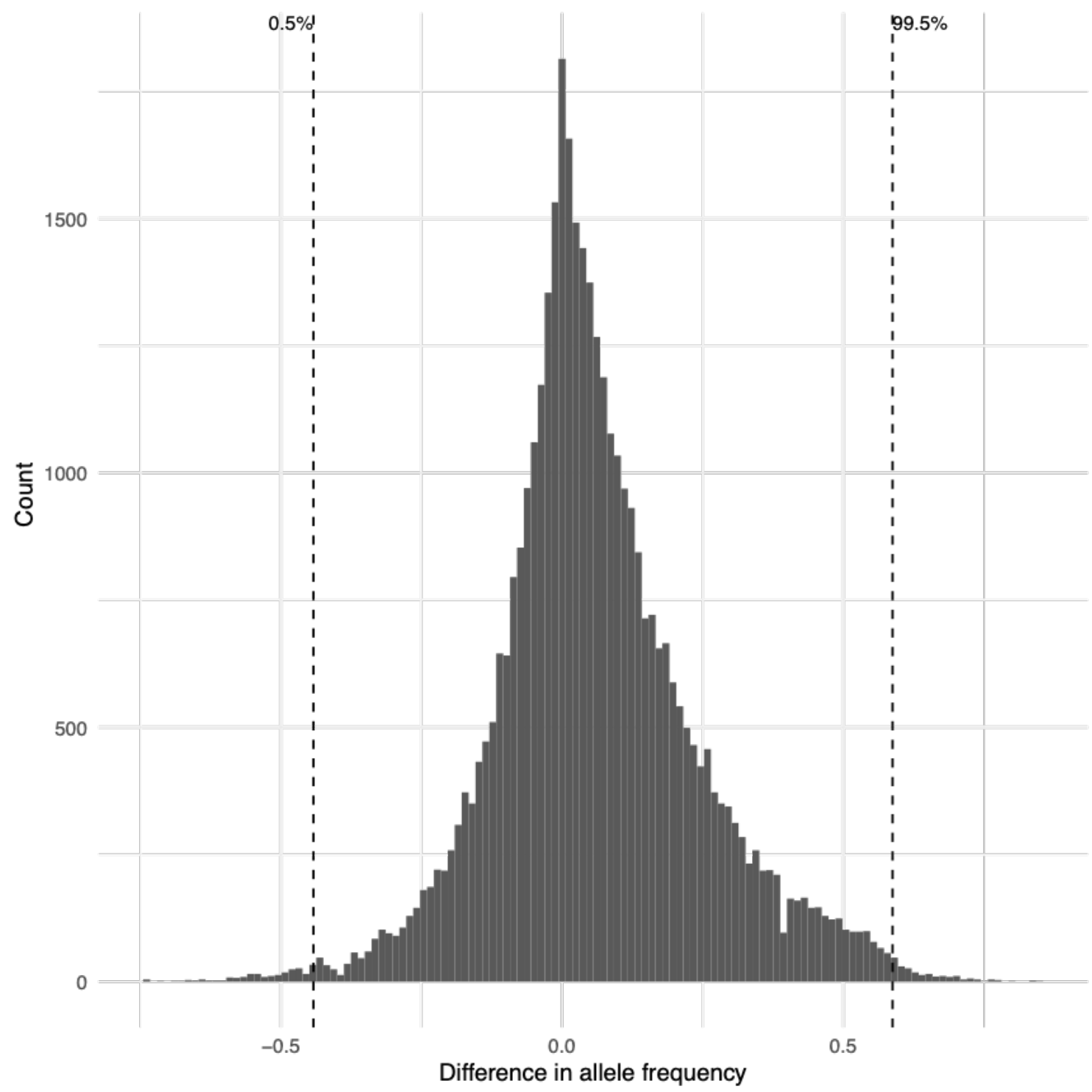

Figure S2: Distribution in difference in allele frequency between sulfidic and nonsulfidic populations, where positive values are higher allele frequency in the sulfidic individuals and negative values are higher allele frequency in the nonsulfidic individuals. Dotted lines represent 99.5 and 0.05 percentile outlier threshold.

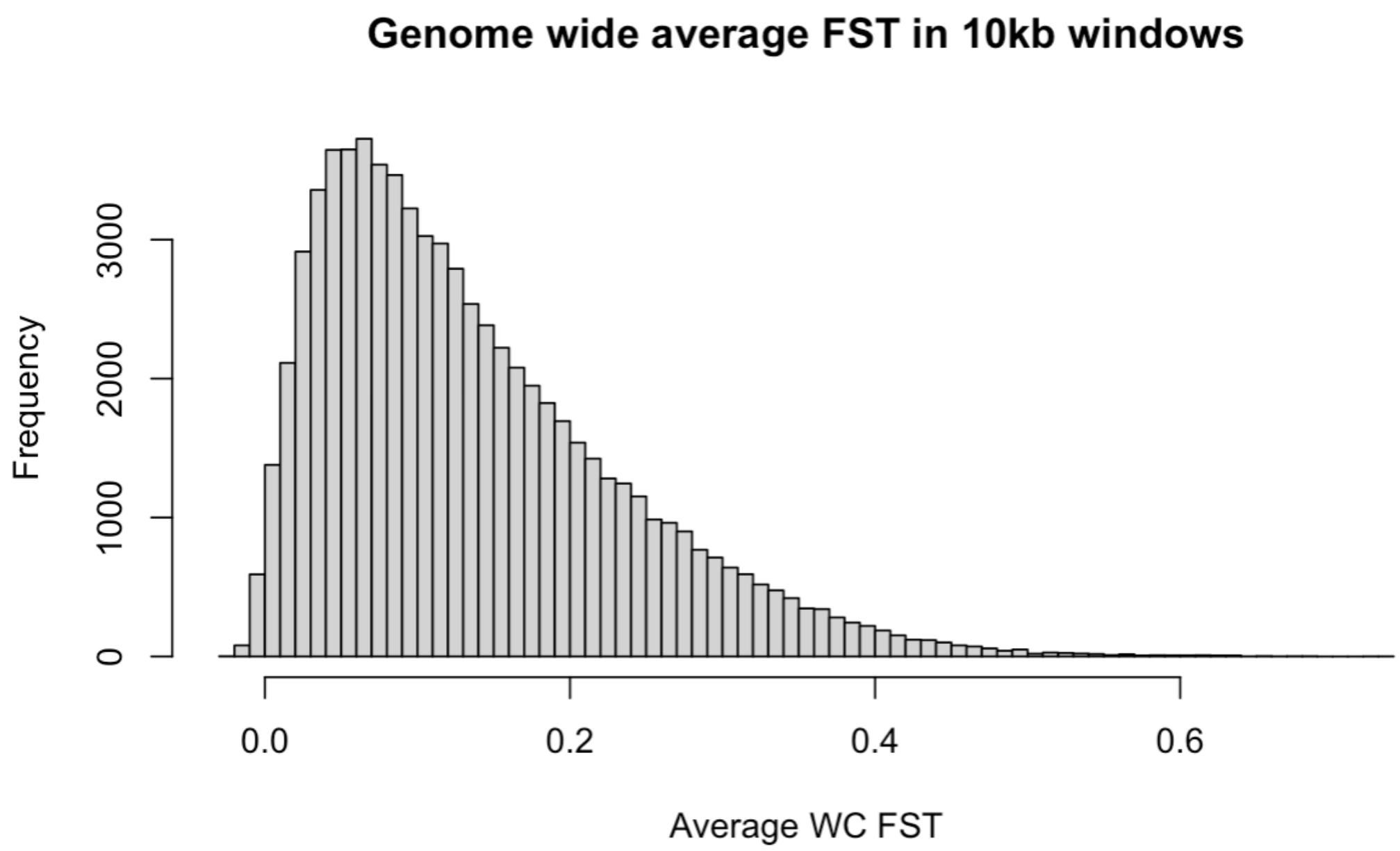

Figure S3: Distribution of weighted average Weir and Cockerham F_{ST} (WC FST) calculated in 10 kilobase (kb) windows across the genome.

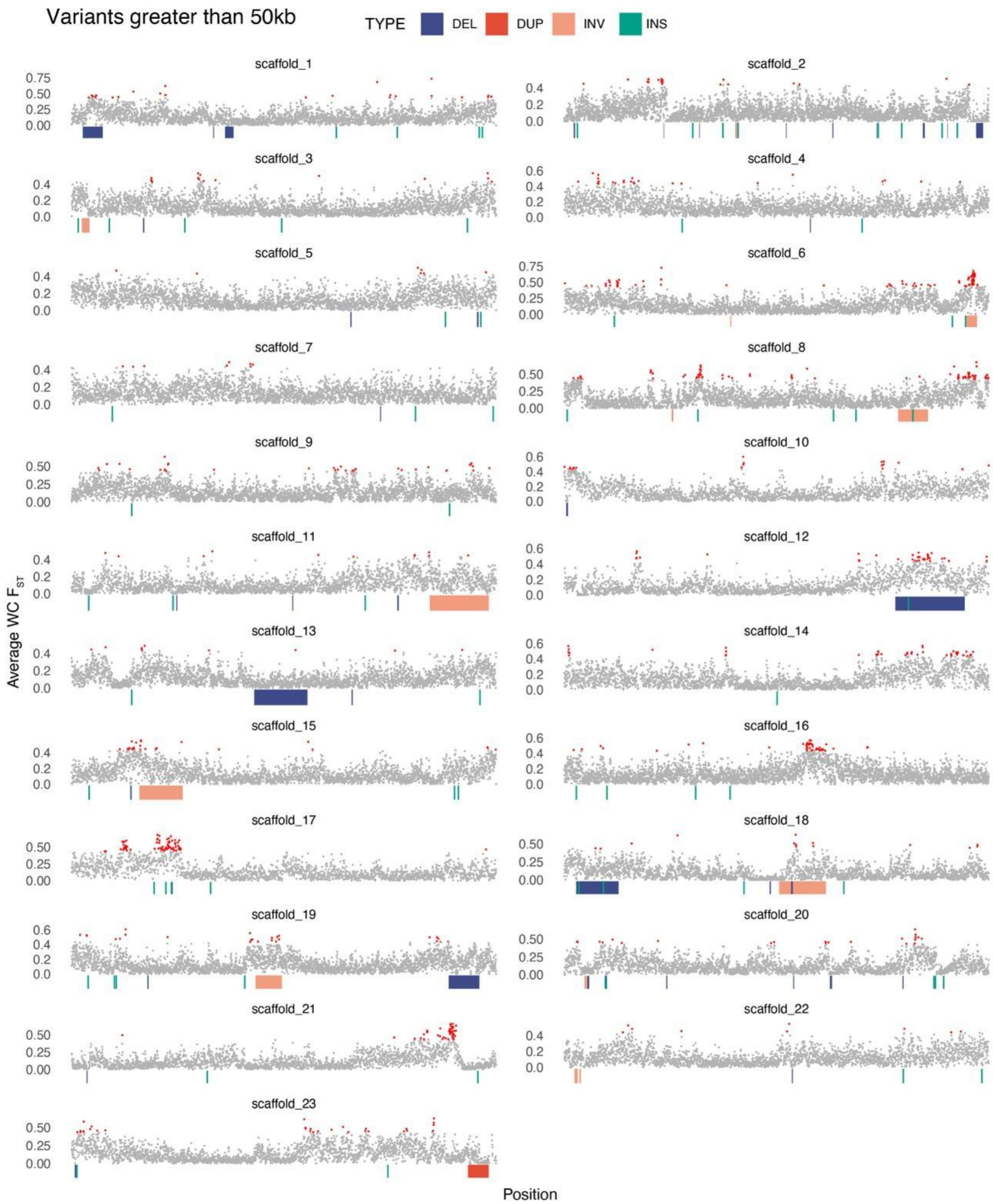

Figure S4: Identification of highly differentiated (F_{ST}) outlier regions between sulfide adapted and nonsulfidic individuals and overlap with large variants greater than 50 kb. Points indicated in red had a Z score > 2.996 and a F_{ST} > 0.431 (0.99 empirical cutoff). Location of variants between 50 kb - 5 Mb plotted below F_{ST} outlier window.

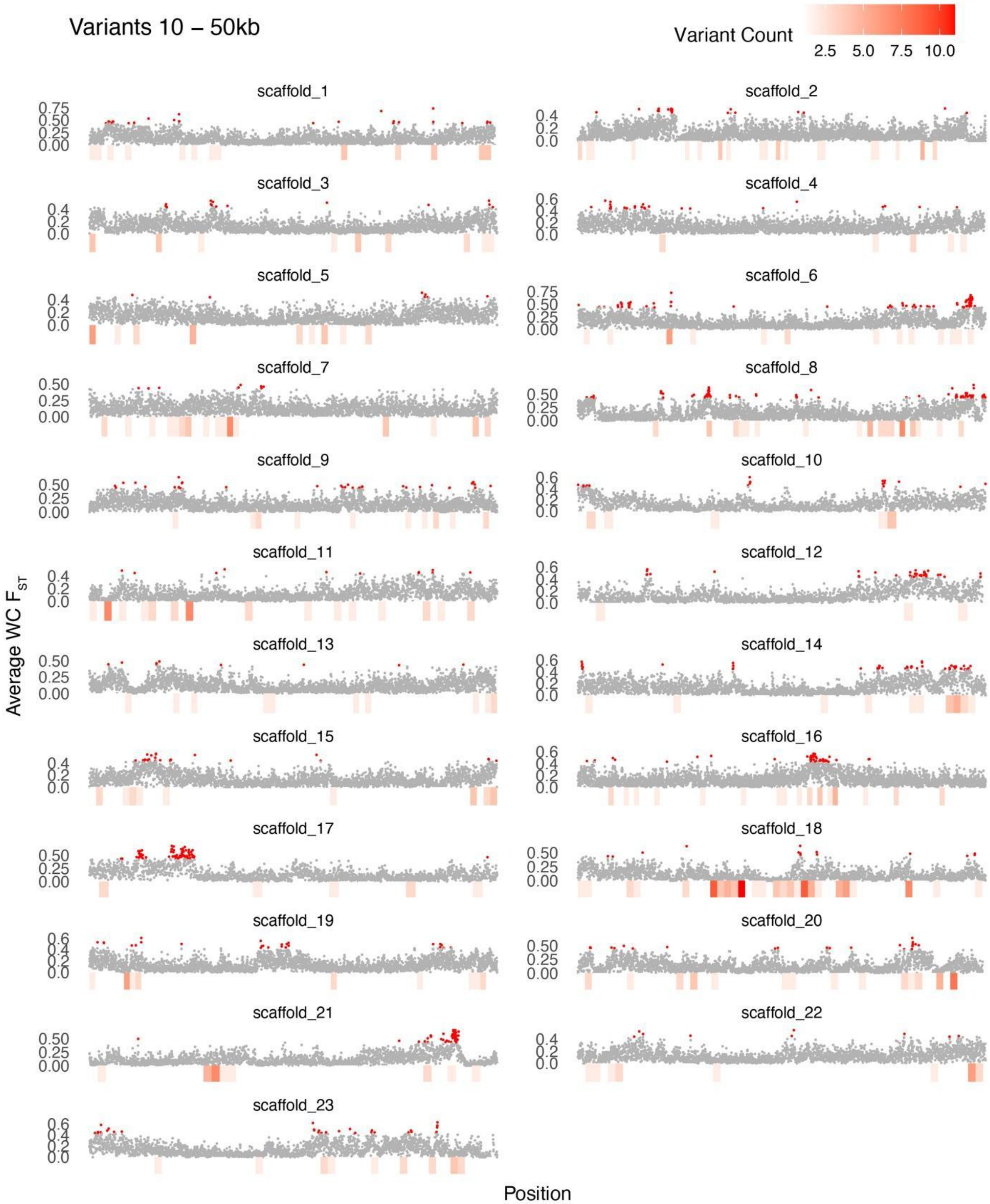

Figure S5: Identification of highly differentiated (F_{ST}) outlier regions between sulfide adapted and nonsulfidic individuals and overlap with variants 10-50kb in size. Points indicated in red had a Z score > 2.996 and a F_{ST} > 0.431 (0.99 empirical cutoff). Heatmap below indicates the quantity of variants within a 500kb window.

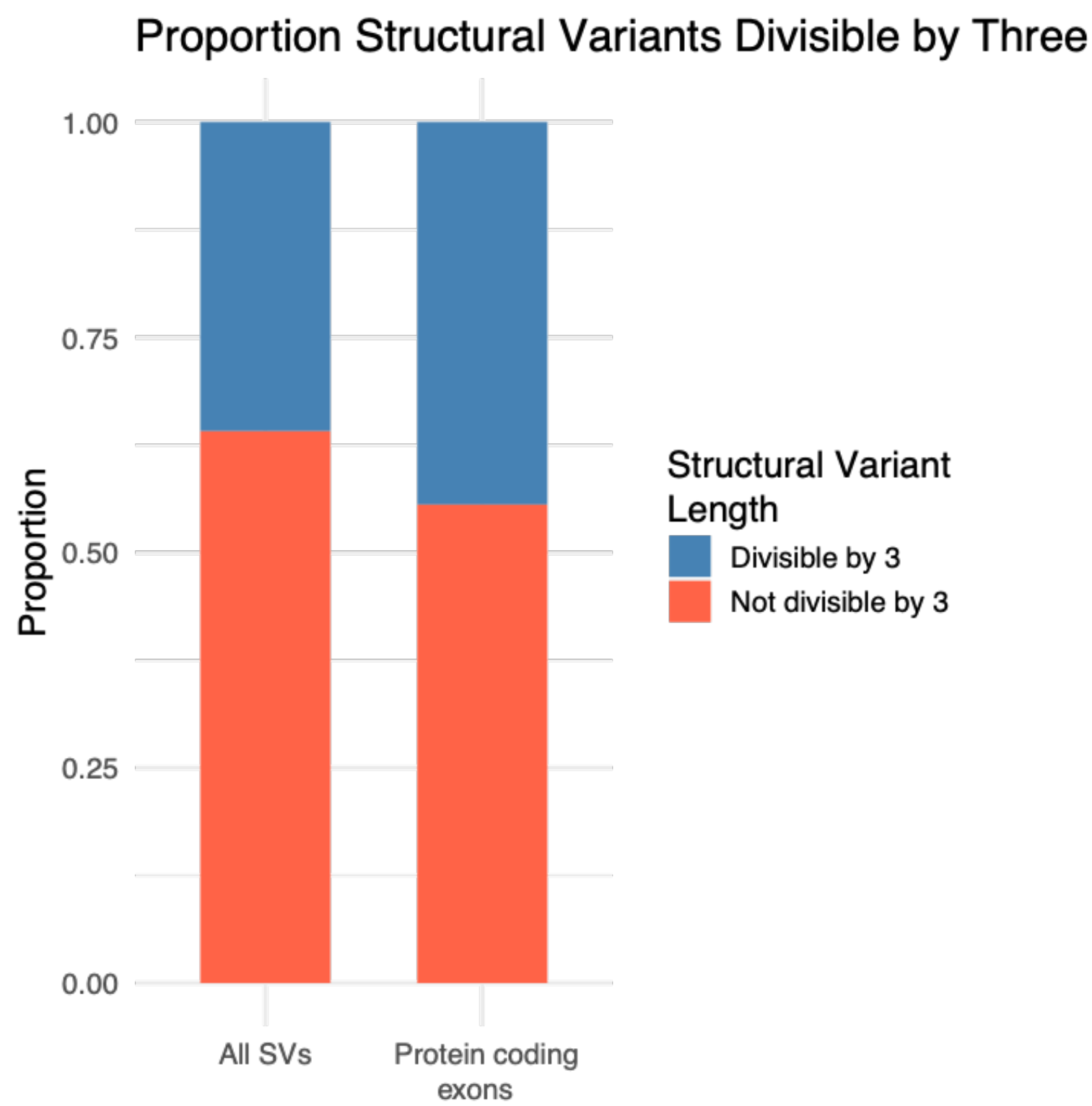

Figure S6: Proportion of in-frame variants (divisible by three) in all variants and in variants found in protein coding exons (blue section of proportion).

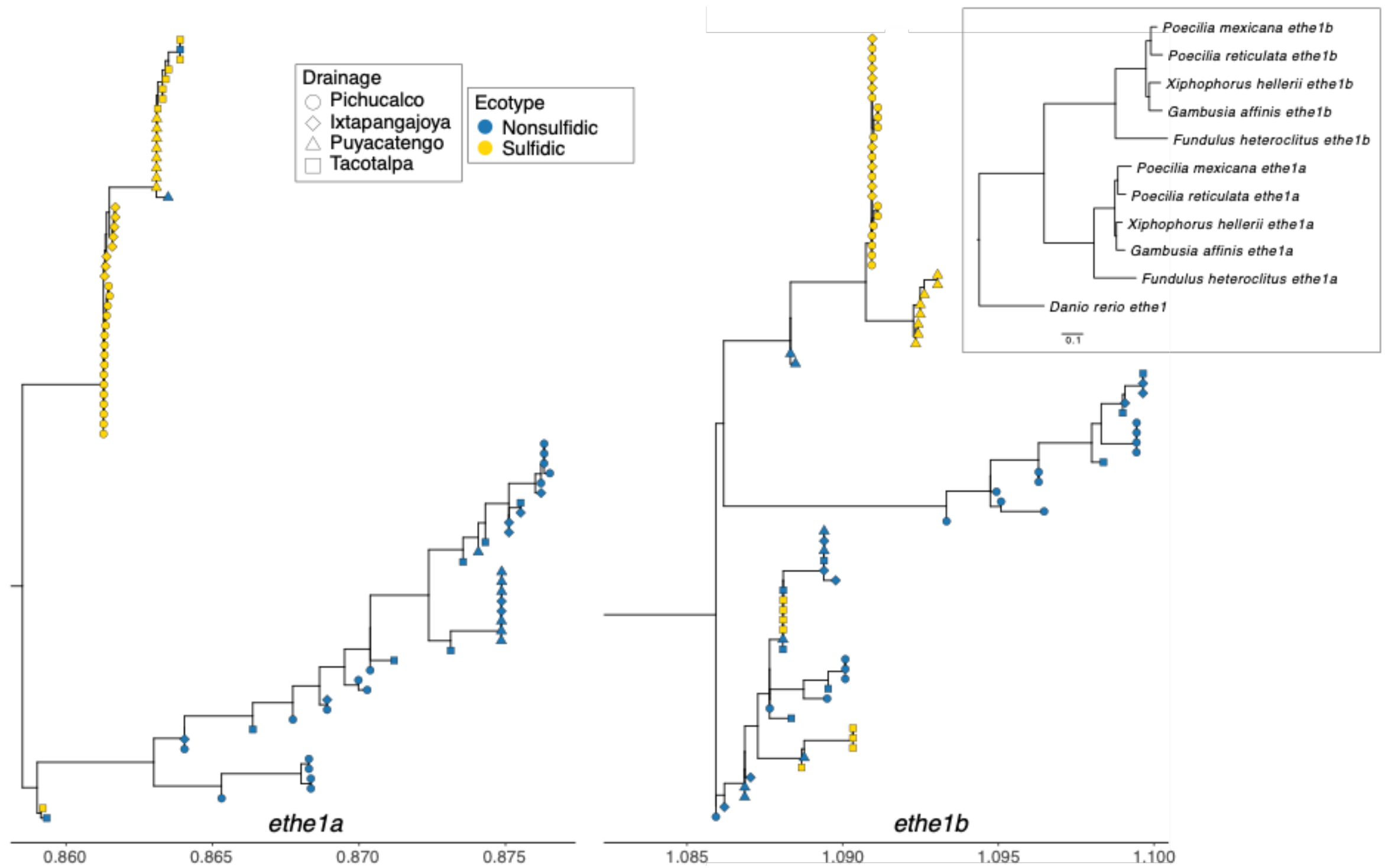

Figure S7: Gene trees for copies of *ethe1* for Poecilia mexicana individuals included in this study. For each tree, color represent the ecotype (yellow - sulfidic, blue - nonsulfidic). Shape represents drainage of origin (Pichulcalo - circle, Ixtapangajoya - diamond, Puyacatengo - triangle, Tacotalpa - square). Additionally, phylogenetic tree of *ethe1* paralogs is shown in the upper right, including a species without the duplication (*Danio rerio*).
